# Disruption of *Mbd5* in mice causes neuronal functional deficits and neurobehavioral abnormalities consistent with 2q23.1 microdeletion syndrome

**DOI:** 10.15252/emmm.201404044

**Published:** 2014-07-07

**Authors:** Vladimir Camarena, Lei Cao, Clemer Abad, Alexander Abrams, Yaima Toledo, Kimi Araki, Masatake Araki, Katherina Walz, Juan I Young

**Affiliations:** 1 Dr. John T. Macdonald Foundation Department of Human Genetics, University of MiamiMiami, FL, USA; 2 John P. Hussman Institute for Human Genomics, Miller School of Medicine, University of MiamiMiami, FL, USA; 3 Institute of Resource Development and Analysis, Kumamoto UniversityKumamoto, Japan

**Keywords:** autistic disorder, intellectual disability, MBD5, mouse model

## Abstract

2q23.1 microdeletion syndrome is characterized by intellectual disability, motor delay, autistic-like behaviors, and a distinctive craniofacial phenotype. All patients carry a partial or total deletion of methyl-CpG-binding domain protein 5 (*MBD5*), suggesting that haploinsufficiency of this gene is responsible for the phenotype. To confirm this hypothesis and to examine the role of MBD5 *in vivo,* we have generated and characterized an *Mbd5* gene-trap mouse model. Our study indicates that the *Mbd5*^*+/*^^*GT*^ mouse model recapitulates most of the hallmark phenotypes observed in 2q23.1 deletion carriers including abnormal social behavior, cognitive impairment, and motor and craniofacial abnormalities. In addition, neuronal cultures uncovered a deficiency in neurite outgrowth. These findings support a causal role of *MBD5* in 2q23.1 microdeletion syndrome and suggest a role for MBD5 in neuronal processes. The *Mbd5*^+/^^GT^ mouse model will advance our understanding of the abnormal brain development underlying the emergence of 2q23.1 deletion-associated behavioral and cognitive symptoms.

**Subject Categories** Genetics, Gene Therapy & Genetic Disease; Neuroscience

## Introduction

Mammalian DNA is post-synthetically modified by the attachment of a methyl group at the 5-position of cytosine. The majority of DNA methylation in vertebrate cells occurs within the CpG sequence where about 70–80% of CpGs are methylated (Lister *et al*, 2009). DNA methylation plays an important role in the control of gene activity either through effects on specific promoter regions or through global mechanisms that affect many genes, ultimately participating in the regulation of tissue-specific gene expression, X chromosome inactivation, genomic imprinting, and transposable element silencing (Jaenisch & Bird, 2003; Bestor & Bourc'his, 2004).

A family of mammalian proteins with the capacity to selectively recognize methylated DNA has been identified via functional or sequence homology methods, constituting primary candidates for the mediation of the DNA methylation outcomes (Hendrich & Bird, 1998). The binding of these proteins to methylated CpGs occurs through a conserved domain of approximately 70 residues, known as the methyl-CpG-binding domain (MBD). Identified members of the family of proteins containing this domain, include MeCP2, MBD1 to MBD6, setdb1 and setdb2, and BAZ2A and BAZ2B (Roloff *et al*, 2003). MBD3, MBD5, and MBD6 are members of this family based on their recognizable MBD, but their binding to methylated DNA has been questioned (Saito & Ishikawa, 2002; Laget *et al*, 2010).

MBD5 contains a PWWP domain in addition to the MBD domain. The PWWP domain is a proline and tryptophan-rich region found in several chromatin factors, some of which were recently shown to use their PWWP domain as a reader for histone marks (Wagner & Carpenter, 2012). Mammalian proteins that contain a PWWP domain include DNMT3A, DNMT3B, bromodomain-containing protein 1 (BRD1), bromodomain and PHD finger-containing protein 1 (BRPF1), 2 (BRPF2) and 3 (BRPF3), and DNA mismatch repair protein MSH6.

*MBD5* was identified as the causal gene for most phenotypes exhibited by 2q23.1 microdeletion syndrome (Jaillard *et al*, 2009; van Bon *et al*, 2010; Williams *et al*, 2010; Talkowski *et al*, 2011; Cukier *et al*, 2012; Motobayashi *et al*, 2012; Noh & Graham, 2012; Bonnet *et al*, 2013; Mullegama *et al*, 2014). Phenotypes reported for 2q23.1 microdeletion syndrome include developmental delay, learning disability, behavioral difficulties such as autistic spectrum disorder or attention-deficit/hyperactivity disorder, seizures, sleep disturbances, ataxia/unusual gait, failure to thrive, and craniofacial abnormalities (Jaillard *et al*, 2009; van Bon *et al*, 2010; Williams *et al*, 2010; Talkowski *et al*, 2011; Cukier *et al*, 2012; Motobayashi *et al*, 2012; Noh & Graham, 2012; Bonnet *et al*, 2013; Mullegama *et al*, 2014). It was estimated that mutations in *MBD5* contribute up to 1% of ASD cases (Talkowski *et al*, 2011).

MBD proteins play a role in diverse processes such as chromatin remodeling (BAZ2A*,* BAZ2B*,* MBD1*,* MBD2*,* MBD3*,* and MeCP2), DNA damage repair (BAZ1A and MBD4), histone methylation (SETDB1 and SETDB2), and X chromosome inactivation (Roloff *et al*, 2003; Bogdanovic & Veenstra, 2009), and their biologic significance is underscored by their involvement in human diseases. The salient example is MeCP2, mutations of which are associated with a progressive neurological disorder, Rett syndrome (RTT), in humans (Amir *et al*, 1999) and RTT-like phenotypes in mice. However, virtually nothing is known about the function of MBD5.

In this paper, we describe a murine model carrying an insertional mutation in *Mbd5* generated through gene-trap mutagenesis. This hypomorphic model recapitulates cardinal phenotypes of 2q23.1 microdeletion syndrome and therefore allows us to investigate the role of MBD5 in brain function, including its role in neuronal maturation. Neurobehavioral phenotypic characterization of the heterozygous mutant mice showed impaired learning and memory, altered social behavior and craniofacial abnormalities, a phenotype that mimics the alterations found in patients. Moreover, we found that MBD5 has transactivational activity and plays a role in neuritogenesis.

## Results

### Transcripts from the *Mbd5* locus

**Figure 1:**
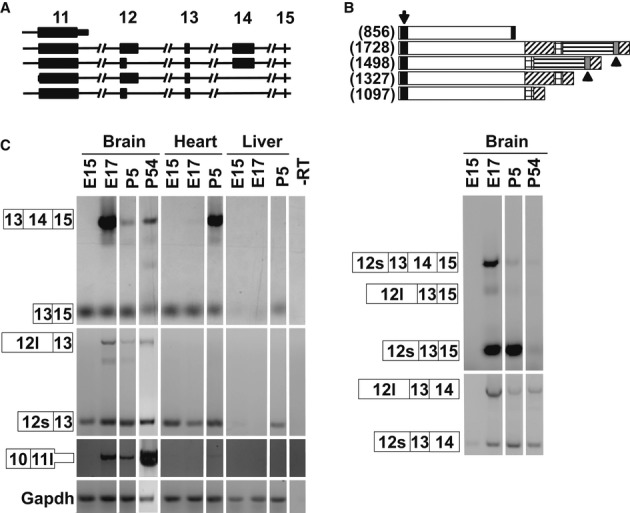
Expression of mouse *Mbd5* transcripts A   Schematic of the *Mbd5* transcripts depicting exons 11–15. B   Schematic of the predicted mouse MBD5 proteins. The black box (arrow) represents the MBD, the gray box (arrowheads) the PWWP domain, and the shaded areas represent alternative regions of the proteins. In brackets is the number of amino acids. C   RT–PCR from cDNA derived from mouse brain, heart, and liver at the indicated developmental time points. The numbered boxes represent the exons included in the amplicons. 12l and 12s depict the extended and short exon 12, respectively; and 11l depicts the extended exon 11. The data show that 11l is a terminal exon since we did not detect transcripts carrying exon 11l spliced with any downstream exon.

Humans and mice contain a single MBD5 gene, located in chromosome 2. Human *MBD5* consists of 10 coding exons (exons 6–15) and 5 upstream noncoding exons. A recent report identified two human *MBD5* transcripts that differ in the inclusion of intron 9, encoding for two protein variants of 1,448 and of 851 amino acids, respectively (Laget *et al*, 2010). Mouse *Mbd5* has 17 exons, 10 of them coding (8–17) and is predicted to express five different RNA isoforms (Fig 1A) that would generate five protein isoforms (Fig 1B). All MBD5 predicted isoforms contain a MBD, but only 2 of them contain a PWWP domain. We analyzed the expression of *Mbd5* isoforms by RT–PCR, followed by confirmatory sequencing, in mouse tissues at different developmental time points. Our results confirmed expression of variants with alternative inclusion of part of intron 11 (corresponding to human intron 9) (Laget *et al*, 2010) and additional transcripts containing either full or part of exon 12 and exclusion or inclusion of exon 14. The relative expression of the different isoforms varies depending on the tissue tested and the developmental stage of the animal, but all of the splice isoforms were present in brain samples (Fig 1C).

### Generation of Mbd5 hypomorph mouse

**Figure 2:**
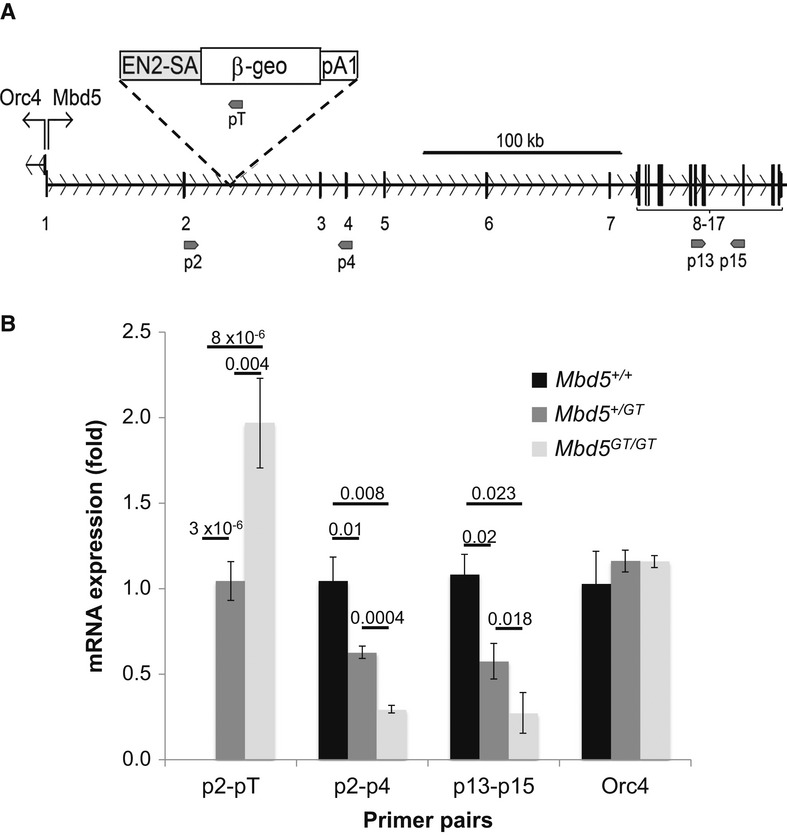
Insertion of a β-geo gene-trap cassette creates an *Mbd5* hypomorph mouse A   Schematic of the insertional mutation of *Mbd5* in intron 2 with the β-geo gene-trap cassette pU-21B. Numbers correspond to the exons of *Mbd5*. Upward arrows indicate the start of the *Orc4* and *Mbd5* genes, respectively. Primers used in (B) are depicted by small arrows. B   Expression analysis of *Mbd5* transcripts was measured by quantitative real-time PCR on E21 mouse brains (*n* = 6 *Mbd5*^+/+^, *n* = 7 *Mbd5*^+/GT^, and *n* = 3 *Mbd5*^*GT/GT*^; Student's *t*-test; unpaired, two-tailed distribution, *P*-values are displayed above the bars in the figure).

Embryonic stem cells from the line Ayu21-B205, containing a single insertion of the pU-21B gene-trap cassette (Taniwaki *et al*, 2005) in intron 2 of the *Mbd5* confirmed by 5′-inverse PCR (Fig 2A and Supplementary Fig S1), were aggregated with ICR morulas to generate chimeras. Chimeric mice were then mated to C57Bl/6, and a founder line was expanded in the C57Bl/6 background for more than six generations. Mice carrying the gene-trap allele are referred to as *Mbd5*^*GT*^.

From *Mbd5*^+/GT^ heterozygous matings, we obtained 165 pups. Twenty pups were found dead within the first 24 h of birth. The genotype of the surviving progeny indicated a ratio of genotypes significantly different from the expected Mendelian transmission for the trapped allele (Fisher's exact test *P* ≤ 0.0001). We obtained 57.1% (*n* = 80) of *Mbd5*^+/GT^ heterozygous, 42.1% (*n* = 59) of wild-type, and 0.7% (*n* = 1) of *Mbd5*^GT/GT^ homozygous genotype. We were able to genotype 8 of the pups that died perinatally and they were all homozygous. One homozygous mouse survived for more than 20 days in a very poor condition and had to be sacrificed for failure to thrive. At E17 and P0, the numbers of *Mbd5*^*GT/GT*^ embryos/newborns did not differ significantly from expected values (6 *Mbd5*^*GT/GT*^, 22 *Mbd5*^*+/GT*^, and 12 wild-type mice; Fisher's exact test *P* = 0.3329). Statistically significant losses were only observed at postnatal stages, suggesting that the majority of the loss was perinatal.

To determine the functional effect of the intronic insertion of the gene-trap cassette on the expression of Mbd5, we checked by RT–PCR whether *Mbd5*^GT^ mice expressed the expected Mbd5-genetrap fused transcript. RNA extracted from wild-type, *Mbd5*^*+/GT*^*,* and *Mbd5*^*GT/GT*^ brain tissue was reverse-transcribed, and RT–PCR was carried out to detect the splicing of Mbd5 exon 2 with the β-geo of the gene-trap cassette. We observed expression of a chimeric mRNA in *Mbd5*^*+/GT*^ and *Mbd5*^*GT/GT*^ mice, absent in wild-type mice (Fig 2B). The level of expression of this Mbd5-genetrap fused transcript was twofold in *Mbd5*^*GT/GT*^, as compared to the *Mbd5*^*+/GT*^ samples, suggesting biallelic expression of the trapped mRNA. This chimeric mRNA, containing untranslated exons 1 and 2 of *Mbd5,* will encode β-geo under the control of the Mbd5 endogenous promoter. However, using primers flanking the region of mRNA interrupted by the gene trap, we detected dose-dependent residual expression of *Mbd5* mRNA in the in *Mbd5*^*+/GT*^ and *Mbd5*^*GT/GT*^ mice, showing that both wild-type and gene-trap fusion products were being produced from the trapped allele (Fig 2B) and suggesting partial use the splice acceptor of the gene-trap cassette. The observation that similar reduction in mRNA abundance due to gene-trap interference was observed by using primers flaking the gene-trap insertion (p2 and p4, Fig 2A) and using primers downstream of the insertion (p13 and p15, Fig 2A) suggest that transcription in the MBD5 locus starts at a single promoter upstream of exon 1.

Thus, these data suggest that the *Mbd5*^*GT/GT*^*-*trapped mice are hypomorphs rather than null Mbd5 mutants and that the inserted β-geo can be utilized as a marker for *Mbd5* endogenous expression.

The *Orc4* gene, shown to be causative for Meier–Gorlin syndrome (Guernsey *et al*, 2011), is located just upstream of *Mbd5* and is transcribed in the opposite direction. Since these two genes might utilize overlapping, or even a single bidirectional promoter (in both humans and mice, there are ESTs for ORC4 and MBD5 that exhibit antisense overlap), we tested whether the insertion of the gene trap affected the expression of *Orc4*. No change in *Orc4* expression was observed in the mutant mice, as compared to the wild-type (Fig 2B), eliminating the possibility of a confounding factor in the study of the gene-targeted mice.

### Mbd5 is highly expressed in neurons

**Figure 3:**
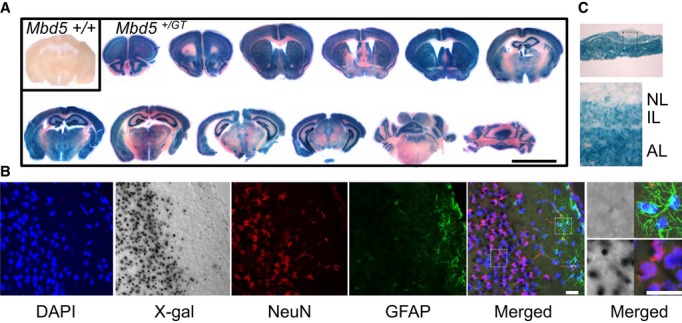
Pattern of expression of mouse *Mbd5* 30-μm cryostat sections from brain (A and B) and pituitary (C) from *Mbd5*^+/+^ and *Mbd5*^+/GT^ mice of 1 month of age were processed for X-gal staining (*n* = 2 *Mbd5*^+/+^, *n* = 4 *Mbd5*^+/GT^). A   Brain sections. Scale bar = 5 mm. B   Sections were also subjected to immunofluorescence for the detection of NeuN (red) and GFAP (green). Nuclei were stained with DAPI, and the images visualized using a confocal microscope. Transmittance image (X-gal) and fluorescent images from DAPI (blue), NueN (red), GFAP (green), and merged fluorescence (X-gal/DAPI/GFAP/NeuN) obtained by confocal acquisition. Magnified regions containing GFAP^+^ astrocytes and NeuN^+^ neurons are shown in the right panel to highlight the elevated β-gal activity in neuronal cells. Scale bar = 50 μm. C   X-gal staining of 30-μm pituitary sections showing expression in the intermediate (IL) and anterior (AL) lobes.

To determine the pattern of expression of the *Mbd5* gene with cellular resolution, we performed X-gal staining on frozen sections prepared from the brains of adult *Mbd5*^*+/GT*^ mice. Positive staining indicative of β-galactosidase expression from the *Mbd5* locus was detected throughout the brain at high levels, predominantly in the cortex, olfactory bulb, striatum, hippocampus, and cerebellum (Fig 3A). In the cortex and striatum, the expression was observed in all areas. In the olfactory bulb, it was higher in the granular cell layer, mitral cell layer, and anterior olfactory nuclei, and in the hippocampus, the expression was observed in areas CA1, CA2, CA3, and the dentate gyrus (Fig 3A). In the cerebellum, the expression was higher in the granular layer. Other sites of strong expression include the thalamus and hypothalamus (Fig 3A).

To examine the cellular identity of *Mbd5-*expressing cells, we performed co-staining with X-gal as an *Mbd5* surrogate, anti-NeuN as a neuronal marker, and anti-GFAP to label astrocytes. We found strong X-gal staining in NeuN-positive cells and no or little X-gal staining in GFAP-positive cells, suggesting that *Mbd5* is expressed predominantly in neurons (Fig 3B).

In addition to the brain, high expression of Mbd5 in adult mice was also observed in the anterior and intermediate lobes of the pituitary (Fig 3C), the heart, and the kidney; and lower levels in the lung, spleen, intestine, and liver (Supplementary Fig S2A).

During embryonic development, we observed positive β-gal activity as early as 5 days post-coitum (Supplementary Fig S2B). At E15.5, we observed strong expression in the developing central nervous system, forebrain, midbrain, hindbrain and spinal cord, and in the heart (Supplementary Fig S2B).

### *Mbd5*^*+/GT*^ mice are smaller and display craniofacial and motor abnormalities

**Figure 4:**
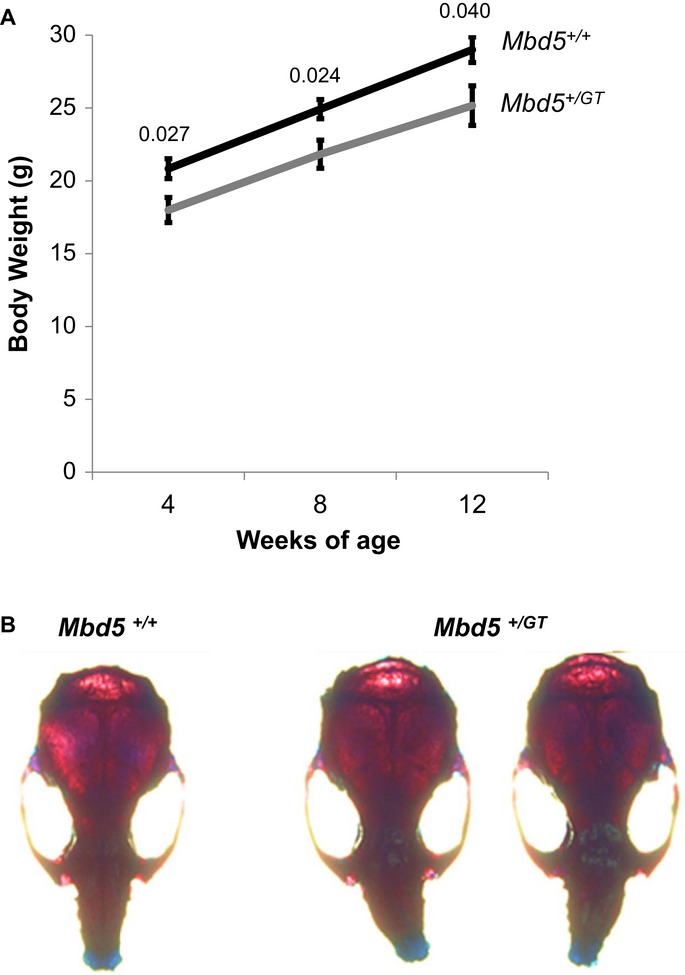
Reduced body weight and craniofacial abnormalities in *Mbd5*^+/GT^ mice A   Body weight of male *Mbd5*^+/+^ and *Mbd5*^+/GT^ mice measured biweekly from 4 to 8 weeks of age differ significantly (*n* = 9 *Mbd5*^*+/+*^ and *n* = 13 *Mbd5*^*+/GT*^; Student's *t*-test; unpaired, two-tailed distribution, *P*-values are displayed above the bars in the figure). B   Top view from skulls of male *Mbd5*^+/+^ and *Mbd5*^+/GT^ mice at 1 month of age.

To investigate the phenotypic consequences of Mbd5 deficiency, we analyzed the phenotype of the *Mbd5*^*+/GT*^ mice. *Mbd5*^*+/GT*^ mice exhibited normal coat condition, presence of full whiskers, and reflexes in the normal range, but were found to be smaller than their wild-type littermates throughout life (Fig 4A). Body weight was significantly reduced in *Mbd5*^*+/GT*^ mice (*P *=* *0.026, Fig 4A). This difference seems to be mostly due to a reduction in abdominal fat (*P* = 0.02, Supplementary Fig S3A), since no other organ was found to be significantly different when compared to wild-type littermates. Craniofacial abnormalities were noted in approximately 60% of *Mbd5*^*+/GT*^ mice (Fig 4B), mainly because of a deviation of the snout resulting from an abnormal nasal bone (Supplementary S3B).

**Figure 5:**
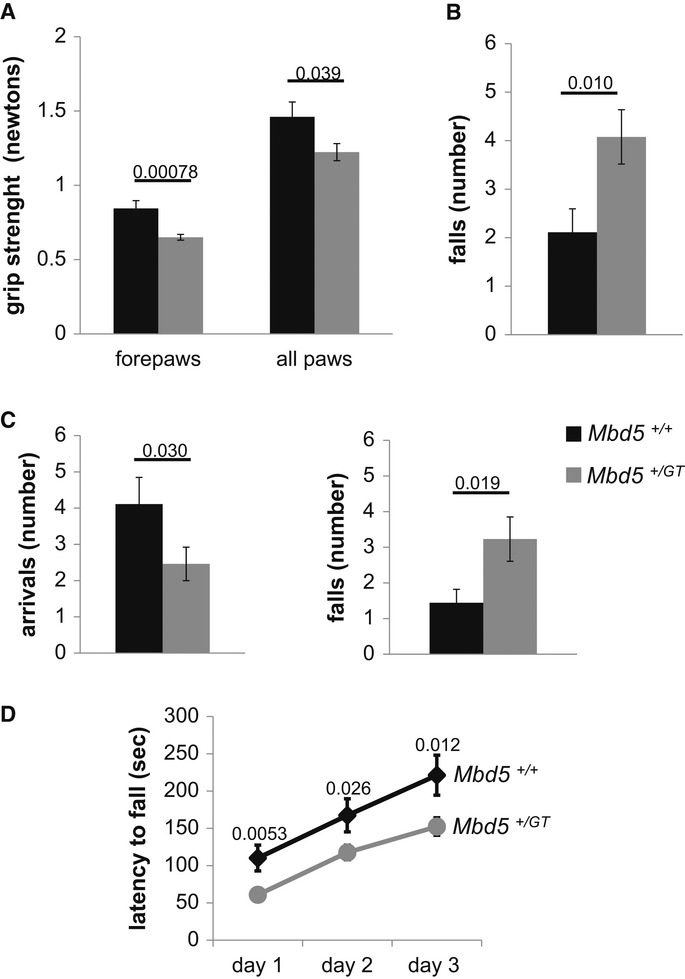
*Mbd5*^+/GT^ mice display abnormalities in motor performance A   *Mbd5*^+/GT^ mice have decreased grip strength than *Mbd5*^+/+^controls. B   *Mbd5*^+/GT^ mice were less capable than *Mbd5*^+/+^ mice to hang from an elevated wire with their forepaws. C *Mbd5*^+/GT^ displayed impaired motor coordination compared with *Mbd5*^+/+^ mice on the dowel test. *Mbd5*^+/GT^ mice reach the platform less times (arrivals) and fell of the dowel more frequently than *Mbd5*^+/+^ mice. D   Performance on the accelerating rotarod is deficient in *Mbd5*^+/GT^ compared with *Mbd5*^+/+^ over all trials (6-week-old male mice, *n* = 9 *Mbd5*^*+/+*^ and *n* = 16 *Mbd5*^*+/GT*^; Student's *t*-test; unpaired, two-tailed distribution, *P*-values are displayed above the bars in the figure).

We tested the neuromuscular strength of the mutant mice, as compared with their wild-type littermates by utilizing a grip strength meter. *Mbd5*^*+/GT*^ mice had significantly less strength than the wild-type controls (*P* = 0.036, Fig 5A). To confirm these results of reduced strength, we compared the capacity of the mice to remain suspended from an elevated wire with their forepaws. This test revealed that *Mbd5*^*+/GT*^ mice were less capable of hanging from the wire than wild-type mice (*P* = 0.01, Fig 5B), supporting the finding of reduced forepaw strength in these mice.

To test for motor coordination, we analyzed the ability of mice to walk on a thin, horizontal wooden dowel. *Mbd5*^*+/GT*^ mice lost their balance and fell off the dowel more frequently that their wild-type littermates (*P* = 0.019, Fig 5C). This motor deficit was further confirmed by testing the mice on the rotating rod apparatus (rotarod). The capacity of the *Mbd5*^*+/GT*^ to remain on the accelerating rotating rod was significantly affected, as compared to the WT mice in all the 3 days of test (*P* < 0.05, Fig 5D), indicative of a motor defect.

### Normal levels of activity and anxiety were observed in *Mbd5*^*+/GT*^ mice

Novel environment-induced activity and anxiety were tested to further characterize the neurobehavioral phenotype of *Mbd5*^*+/GT*^ mice. We utilized the open-field test to study both, exploratory activity and anxiety-related responses in a novel arena as previously described (Walz *et al*, 2004). Analysis of the open-field data showed no significant differences in horizontal activity levels; total traveled distance was 58.2 ± 5.9 m and 63.1 ± 5.1 m for *Mbd5*^*+/GT*^ and wild-type mice, respectively (*P* = 0.57, Supplementary Fig S4A). Anxiety levels can be estimated by comparing the time the mice explore the most exposed center area of the open field versus the peripheral area. No significant difference was observed in this parameter, suggesting a normal level of anxiety for the mutant mice (*P* = 0.96, Supplementary Fig S4B). In addition, no differences were found between the *Mbd5*^*+/GT*^ and the *Mbd5*^*+/+*^ mice in the plus maze, a more sensitive test to evaluate anxiety levels (Supplementary Fig S4C), further supporting the conclusion of normal anxiety in the mutant mice.

### *Mbd5*^*+/GT*^ mice showed abnormal social behavior and impaired learning and memory

**Figure 6:**
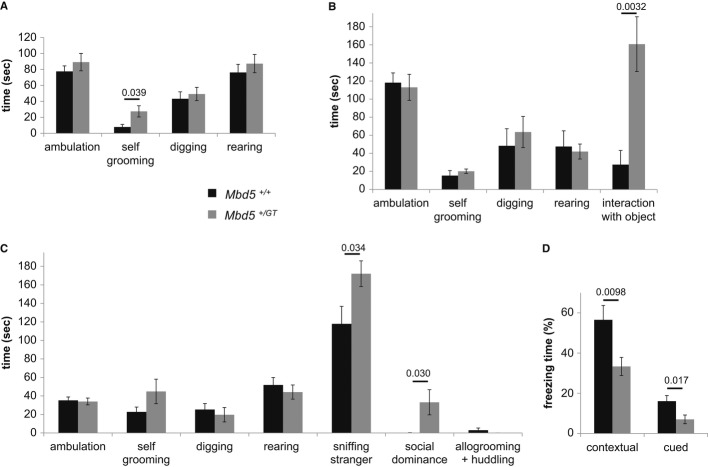
Abnormal social behavior and deficient learning in *Mbd5*^+/GT^ mice A–C   Evaluation of activities of male mice observed over 10 min in their own cage. Bars represent total time mice spent in the indicated activity. (A) Observation of unperturbed mice. (B) Evaluation of mice in the presence of a novel object (Eppendorf 1.5-ml tube). (C) Social interaction of male test mice in the presence of a stranger mouse. D   *Mbd5*^+/GT^ mice show impaired contextual and cued conditioning. Bars represent the amount of freezing behavior, which is used as an index for recollection of the context or tone. Age of male mice was between 12 and 13 weeks. Asterisks indicate a significant change in *Mbd5*^+/GT^ mice (*n* = 9 *Mbd5*^*+/+*^ and *n* = 14 *Mbd5*^*+/GT*^; Mann–Whitney *U*-test, *P*-values are displayed above the bars in the figure).

We examined the social behavior of the *Mbd5*^*+/GT*^ by using a sensitive test of social interaction that allows for direct inter-individual contact (Egashira *et al*, 2007; Sato *et al*, 2012). The test consists of analysis of behaviors manifested by the test mouse in his own cage in three consecutive 10-min sessions: undisturbed, nonsocial engaging (a novel object is introduced), and social engaging (a non-familiar mouse is introduced). *Mbd5*^*+/GT*^ mice spent three times as much grooming themselves as littermate WT mice during the undisturbed period (Fig 6A). This increase in total time spent grooming was due to a significant increase in the frequency of grooming events (Supplementary Fig S5A). No significant differences were observed in ambulatory time, digging or rearing during the undisturbed session. After the addition of a novel inanimate object, *Mbd5*^*+/GT*^ mice spent significantly more time interacting with the object than WT littermates (30% of the time for the *Mbd5*^*+/GT*^ versus 5% for WT mice, Fig 6B). Notably, the comparatively enhanced activity elicited by the object on the *Mbd5*^*+/GT*^ mice is associated with a normalization in self-grooming (*P* > 0.19, Mann–Whitney *U*-test, Fig 6B). The introduction of a presumably non-intimidating, same sex stranger mice in the cage revealed abnormal social behavior of the *Mbd5*^*+/GT*^ mice; they spent significantly more time interacting with the stranger than WT mice. Time spent in neutral social interactions like sniffing the stranger (*P* < 0.05, Mann–Whitney *U*-test Fig 6C), as well as in socially dominant interactions such as mounting and fighting (*P* < 0.05, Mann–Whitney *U*-test Fig 6C) was elevated in *Mbd5*^*+/GT*^ mice. A trend toward an increase in self-grooming activities in the *Mbd5*^*+/GT*^ mice did not reach statistical significance (Fig 6C). Because olfactory cues are important mediators of normal social interactions, we tested whether olfaction was normal in *Mbd5*^*+/GT*^ mice. Slightly fasted mice of the two genotypes displayed the same latency in finding buried food (*P *>* *0.05, Supplementary Fig S5B).

We then examined emotional learning and memory processes in the *Mbd5*^*+/GT*^ mice by fear conditioning. In this test, responses are measured by freezing, which can be elicited by associating a neutral cue (e.g., environmental context or tone) with an aversive event (e.g. electric foot-shock) (Johansen *et al*, 2011; Maren *et al*, 2013). Mice were evaluated 24 h after conditioning, and we found that freezing was significantly reduced in the *Mbd5*^*+/GT*^ mutant mice in both the context and the cue tests (*P *=* *0.009 and *P *=* *0.017, respectively, Fig 6D). Importantly, sensitivity to foot-shock was not distinguished by genotype. Hence, *Mbd5*^*+/GT*^ hypomorph mice are impaired in contextual and cued fear conditioning.

### Reduced neurite outgrowth in *Mbd5*^*+/GT*^ neurons

Our behavioral experiments strongly support a role for Mbd5 in neuronal function. The presence in MBD5 of a conserved PWWP domain, a motif found in eukaryotic nuclear proteins that participate in cellular differentiation through epigenetic control of gene expression, suggested to us that reduced Mbd5 expression may affect neuronal development ultimately resulting in phenotypic alterations.

**Figure 7:**
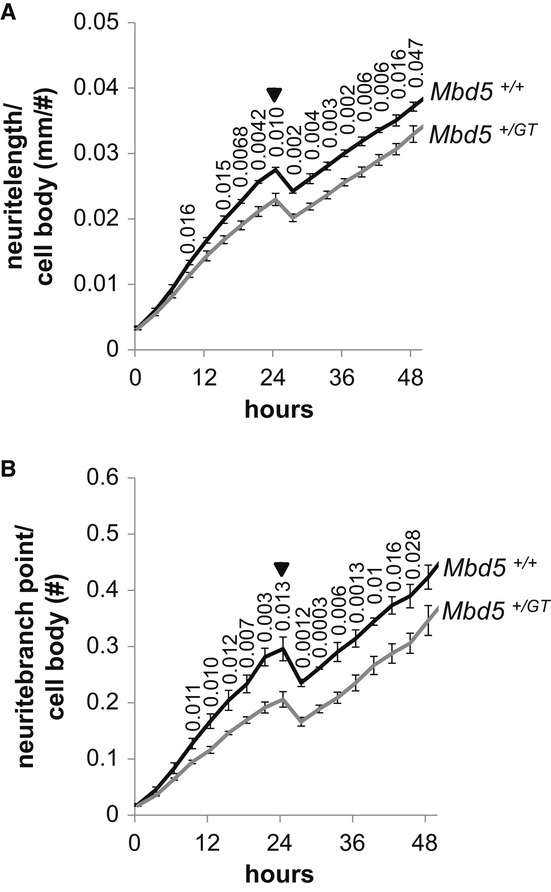
*Mbd5*^+/GT^ neurons have reduced neurite outgrowth and branching A, B   Dissociated E16 cortical neuron cultures generated from *Mbd5*^+/GT^ and *Mbd5*^+/+^ mice were plated, and cellular morphology was recorded every 3 h for 48 h using an IncuCyte live-cell imaging system located within the incubator. Phase images were analyzed for process length and branching by Incucyte's NeuroTrack software. Shown are average values for neurite length (A) and number of branching points (B) normalized to cell bodies of quadruplicate wells per embryo (*n* = 3 *Mbd5*^*+/+*^ and *n* = 4 *Mbd5*^*+/GT*^; Student's *t*-test; unpaired, two-tailed distribution, *P*-values are displayed above the line in the figure). Arrows indicate the removal of the plates from the incubator at 24 h to replace plating media for neurobasal media.

We set out to determine whether the phenotypic alterations were accompanied by a detectable cellular phenotype. To test this suggestion, we analyzed neurite extension in cultured cortical neurons isolated from embryonic day 16 (E16) embryos of *Mbd5*^*+/GT*^ and *Mbd5*^*+/+*^ genotype. Time-lapse microscopy was carried out using the IncuCyte live-cell imaging system, an automated imaging platform that provides real-time images and quantitative data generated throughout 48 h *in vitro*. In comparison with WT neurons, neurite length of *Mbd5*^*+/GT*^ neurons was significantly reduced by 6 h and this reduction remained significant during the first 2 days in culture, suggesting a defect in neurite outgrowth. Since it has been suggested that culture density might affect neurite extension, we normalized the neurite length data with cell number and obtained essentially the same results (Fig 7A). The reduction in neurite length was accompanied by a significant reduction in branching points (Fig 7B). These data suggest that Mbd5 plays an important role in neurite outgrowth and branching.

### MBD5 acts *in vitro* as a transcriptional activator

**Figure 8:**
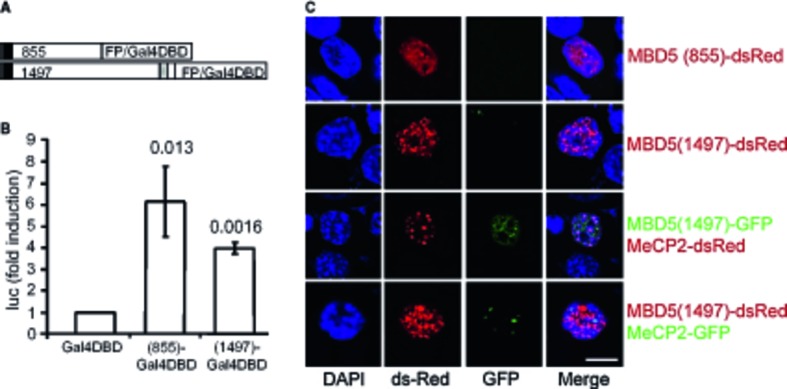
Mbd5 acts *in vitro* as a transcriptional activator A   Schematic of the constructs for the transactivation studies. The black box represents the MBD, and the gray box the PWWP domain. B   Constructs encoding the Gal4 DNA-binding domain fused to Mbd5 and the Gal4-responsive luciferase reporter gene were co-transfected into HEK293 cells. A β-galactosidase expression plasmid was also co-transfected to allow normalization for transfection efficiency. Cells were collected, and luciferase activity was measured 24 h after transfection. Each bar represents the mean ± SD from three independent experiments. (Student's *t*-test; unpaired, two-tailed distribution compared with cells transfected with GAL4-DBD, *P*-values are displayed above the bars in the figure.) C   Localization of transfected Mbd5 in mouse neuroblastoma N2A cells. Mbd5 and the heterochromatic marker MeCP2 fused to GFP or dsRed at the N-terminus were transfected into N2A cells. DAPI was used for nucleic acid staining (blue). The images were taken with a confocal LSM 710 microscope from Zeiss. Scale bar = 10 μm.

Since MBD proteins modulate gene expression, we tested the ability of Mbd5 to modulate transcription in an *in vitro* system. Mouse Mbd5 cDNAs were fused to GAL4 DNA-binding domain (Gal4DBD) and assayed by transient transfection for activation of firefly luciferase activity driven by a GAL4-dependent reporter gene (Fig 8A). Mbd5 (1-1497)-Gal4DBD exhibited transcriptional activation capacity, as well as the shorter Mbd5 (1-855)-Gal4DBD (Fig 8B). It was recently reported that human MDB5 isoforms have nuclear localization in transfected cells. We confirmed nuclear localization of mouse Mbd5 isoforms by fusing them (1–855 and 1–1,497) to fluorescent proteins and determining its localization in transfected mouse (N2A) and human (HEK293) cells. We observed that the 1–855 isoform was diffused throughout the nucleus while the 1–1,497 protein showed a punctuated pattern (Fig 8C). Laget *et al* reported that human MBD5 formed nuclear puncta when transfected into mouse cells that coincided with mouse heterochromatin foci (Laget *et al*, 2010). However, we observed Mbd5 exclusion from heterochromatic foci (Fig 8C). To further test whether Mbd5 localizes to heterochromatin, we co-transfected N2A and NIH-3T3 cells with Mbd5 along with MeCP2, which has a bona fide heterochromatic localization. We observed mostly non-overlapping localization of these two proteins in both cells types, suggesting that mouse Mbd5 does not bind heterochromatin in cultured cells (Fig 8C and Supplementary Fig S6). These data suggest distinct functions for Mbd5 and MeCP2, in spite of the presence of MBD in both proteins.

## Discussion

Patients with 2q23.1 microdeletion syndrome present with intellectual disability, epilepsy, motor delay, and autism features. Although the 2q23.1 locus contains several genes associated with genetic disorders such as ORC4, KIF5C, MMADHC, NEM2, and CACNB4, extensive analysis of the alignment of deleted regions in patients has identified *MBD5* as the only gene included in the smallest region of overlap, suggesting that genetic alterations in this gene cause the primary features of 2q23.1 microdeletion syndrome. The function of MBD5 is virtually unknown, but its involvement in 2q23.1 microdeletion syndrome suggests a critical role in neurodevelopment (Jaillard *et al*, 2009; van Bon *et al*, 2010; Williams *et al*, 2010; Talkowski *et al*, 2011; Cukier *et al*, 2012; Motobayashi *et al*, 2012; Noh & Graham, 2012; Bonnet *et al*, 2013; Mullegama *et al*, 2014).

In addition, microduplications in the same region have been associated with autistic or Angelman-like features and developmental delay, suggesting that either decreases or increases in expression of wild-type MBD5 can result in clinical phenotypes (Chung *et al*, 2011; Bonnet *et al*, 2013; Mullegama *et al*, 2014), further highlighting the relevance of a functional characterization of MBD5.

We describe a mouse model containing a modified *Mbd5* gene that severely decreases Mbd5 expression. In agreement with a recent report of Mbd5-null mice (Du *et al*, 2012), the homozygous *Mbd5*^*GT/GT*^ mutant animals died perinatally. The heterozygous Mbd5-null animals were described as grossly normal; however, they do not report their neurobehavioral phenotype (Du *et al*, 2012). Our functional characterization of the heterozygous *Mbd5*^*+/GT*^ mice suggests that these mice are a construct valid model for 2q23.1 microdeletion syndrome. *Mbd5*^*+/GT*^ mice, which express approximately 60% of endogenous *Mbd5*, recapitulate several features of the human syndrome.

Using behavioral analyses, we observed impairments in fear conditioning and abnormal social interactions in *Mbd5*^*+/GT*^ mice, reminiscent of two of the most characteristic clinical features of the 2q23.1 microdeletion patients, developmental delay/intellectual disability, and autistic-like manifestations. The observed deficit in both hippocampus-dependent contextual fear conditioning and in cued fear memory formation which requires the amygdala suggests an impairment in the general ability to learn basic associations in the *Mbd5*^*+/GT*^ mice that might stem from cortical networks modulating hippocampal and amygdalar functions (Phillips & LeDoux, 1992; Tronson *et al*, 2012). We also observed increased self-grooming, a stereotyped behavior in mice that is often compared to the restrictive repetitive and stereotyped patterns of behavior that are among the diagnostic criteria of autism (American Psychiatric Association, 2000). Of interest is the observation that upon introduction of an object into their home cage, the excessive self-grooming of the *Mbd5*^*+/GT*^ mice was replaced by a compulsive interaction with the novel object. This abnormally high interest in nonsocial object exploration was also observed for social engagement, since *Mbd5*^*+/GT*^ mice spent more time interacting with stranger mice than WT littermates. The exaggerated social engagement of these mice—abnormal not only in its persistence but also in its characteristics (increased dominance behaviors)—is most probably another reflection of the insistence on sameness exhibited by the *Mbd5*^*+/GT*^ mice.

The majority of 2q23.1 microdeletion syndrome patients have diminished motor skills (Jaillard *et al*, 2009; van Bon *et al* 2010; Chung *et al*, 2011; Talkowski *et al*, 2011; Chung *et al*, 2011; Noh & Graham, 2012), a phenotype also exhibited by the *Mbd5*^*+/GT*^ mice. Analysis of motor abilities in the *Mbd5*^*+/GT*^ mice uncovered alterations in strength, balance, and movement coordination. Like 2q23.1 microdeletion syndrome patients, *Mbd5*^*+/GT*^ mice present craniofacial abnormalities evidenced by a deviation of the snout. The fact that we observed deviation toward the left side in approximately half of the mice whereas the other half deviated to the right suggests that the deviated phenotype might result from delayed ossification influenced by interaction with the environment. This could explain why the craniofacial anomalies of the 2q23.1 microdeletion syndrome patients are also inconsistent: For example, the patient's nose has been described as bulbous or prominent, with high nasal bridge, saddle nasal bridge, or small nasal bridge (Williams *et al*, 2010; Talkowski *et al*, 2011; Mullegama *et al*, 2014).

To correlate abnormal behavior to neuronal structural deficits, we analyzed neurite extension from cortical neurons in the early period of differentiation. Reduced neurite length and branching points were observed for *Mbd5*^*+/GT*^ neurons, suggesting an important role for MBD5 in hallmark processes of neuronal differentiation such as neurite outgrowth and maturation. This cellular phenotype may represent a potential substrate for developing therapeutic strategies through phenotypic suppression screenings. The role that MBD5 plays in these differentiation processes is unknown, but the presence of MBD and PWWP domains in the protein suggests that transcriptional control may be involved. Supporting this suggestion, we observed that Mbd5 has transcriptional activator functions *in vitro* when fused to a GAL4 DNA-binding domain. Although this activity was not directly evaluated with an endogenous Mbd5 target promoter, our data are in agreement with a recent report (published while this manuscript was under revision), showing that MBD5 directly activates the transcription of the *Fth1* promoter (Tao *et al*, 2014). Future experiments will study the link between the phenotypic manifestations of the *Mbd5*^*+/GT*^ mice and functional and transcriptional alterations in *Mbd5*^*+/GT*^ neurons.

Taken together, these data show that *Mbd5* haploinsufficiency in mice leads to behavioral and learning and memory impairments accompanied by functional deficits in cortical neurons, providing an experimental model for social disturbances and ID in patients with 2q21.3. This mouse model will be valuable for understanding the pathogenesis of developmental brain disorders as well as for studies toward the design of therapeutic approaches.

## Materials and Methods

### Animals

Mice carrying an insertional mutation in the *Mbd5* locus *(*B6;CB-Mbd5Gt(pU-21B)205Imeg) were generated at the Institute of Resource Development and Analysis (IRDA), Kumamoto University, using a gene-trap construct pU-21B randomly inserted in intron 2 of the mouse *Mbd5* gene in embryonic stem cells from the line Ayu21-B205. The construct pU-21B consists of 1.8 kb of an intron and a splice acceptor sequence from the mouse En-2 gene, a promoterless β*-geo* gene containing three stop codons in-frame with the ATG, and a polyadenylation signal (pA), as recently described (Araki *et al*, 2014). The presence of ES cell in which *Mbd5* was trapped by single copy integration was confirmed by 5′-RACE analysis as reported (Araki *et al*, 2014). In addition, fusion of Mbd5 mRNA of the trapped and the reporter gene was checked by RT–PCR using an *Mbd5-*specific primer (p2: TCGGATCCTAACTAAATCAAAATG) and a primer aligning to the trap vector (pT: GTAATGGGATAGGTTACGTTGGTGTAG). Chimeric mice were produced by aggregation of ES cells with 8-cell embryos of ICR mice (Nippon Clea). Chimeric male mice were mated with C57BL/6 females to obtain F1 heterozygotes, which were then backcrossed with C57BL/6 mice at the University of Miami. Mice were genotyped by PCR. Briefly, DNA fragments were amplified using primers mbd5F: TGTAGGAAAATGTTCAGTCCTGT and either mbd5R: TTTCATTCTGGTAAGAGCCA (for the wild-type) or trapvectorR: CCTGGTGAGGCCAAGTTTGTTTCC (mutant allele) and a protocol of 95°C for 5 min, then 35 cycles of 95°C for 30 s, 55°C if using mbd5R primer or 63°C if using the trapvectorR primer for 30 s, 72°C for 2 min., then a final 72°C for 7 min. PCR products were visualized on agarose gels.

At weaning age, *Mbd5*^*+/GT*^ and wild-type littermate control male mice were housed 2–5 per cage in a room with a 12-h light/dark cycle (lights on at 6 am, off at 6 pm) with access to food and water *ad libitum*. Behavioral testing was performed between 9 am and 6 pm. All behavioral testing procedures were approved by the UM Institutional Animal Care and followed the NIH Guidelines, ‘Using Animals in Intramural Research’.

### Expression analysis and quantitative RT–PCR

RNA was isolated from TRIzol (Invitrogen) homogenized tissue or cells and purified with the RNeasy kit (Qiagen). RT–PCR was performed as described previously (Young *et al*, 2005). We verified that our cDNA preparations were not contaminated by genomic DNA by performing qPCR in the absence of reverse transcription. RT–PCR primer sequences and the expected fragment sizes can be found in Supplementary Table S1.

Quantitative real-time RT–PCR amplifications were performed in triplicate from 50 ng of cDNA using the SYBR Green real-time PCR method on a Roche LightCycler 480 in a total volume of 10 μl, each reaction containing 1 μl of diluted cDNA. The results were analyzed with the LightCycler 480 software, and all values were normalized to the levels of the GAPDH mRNA using the 2̂-(ΔΔ*C*_t_) method. Primer sequences are provided in Supplementary Table S1.

### Behavioral testing

#### Test animals

Male mice of the C57BL/6J genetic background were used for all behavioral analyses. Behavioral testing started when mice were 6 weeks of age. For each test, the number of mice (*N*) tested is indicated in the respective figure caption. Before each test, the mice were placed in the testing room for a habituation period of 30 min. Mouse behaviors were measured with at least 1 day in between tests. Starting at 6 weeks of age, we tested: (i) general characteristics and neurological reflexes, (ii) strength test, (iii) wire hanging test, (iv) dowel test, and (v) rotarod test. At 9 weeks of age, we tested: (vi) open field and (vii) plus maze. At 11 weeks of age, we performed (ix) fear conditioning test, and at 13 weeks of age (x) direct interaction test.

#### General characteristics and reflexes

Mice were evaluated for general health, including body weight and length, appearance of fur and whiskers, reflexive reactions to a gentle touch from a cotton swab to the whiskers and the visual placing reflex.

#### Dowel test

The apparatus consists of two elevated platforms (50 cm from the floor) connected by a wooden dowel. The test was divided into three parts. First, the mice were placed on each platform for 1 min for them to feel the safety of the platform. After that, mice were taken and placed on the wooden dowel 10 cm away from one of the platforms, and the number of falls before reaching the platform within the first min was recorded. The mice that reached the platform in <1 min were placed on the center of the elevated dowel at 30 cm from the platform, and the latency time to reach the platform, the number of arrivals, and the number of falls were recorded during 90 s.

#### Rotarod test

Mice were placed on the accelerating rotarod apparatus (Ugo Basile) for 16 trials (four trials on four consecutive days) with a 20-min rest interval between trials. Each trial lasted for a maximum of 600 s, during which the rod accelerated linearly from 4 to 40 rpm. The amount of time for each mouse to fall from the rod was recorded for each trial.

#### Open-field test

Mice were placed in the center of an open-field apparatus (40 × 40 × 30 cm, EMV-510 Med Associate INC) and allowed to explore for 30 min. Activity in the open field is quantified by a computer-operated activity monitor program from Med Associates system. Total distance, movement time, movement speed, vertical activity, and time in center arena were recorded.

#### Plus maze

Mice were placed in the center of a cross-shaped maze elevated 45 cm from the floor with two open and two closed arms. The behavior of the mice was observed for 5 min, and the time spent in either the closed or open arm or in the center of the maze was recorded. Mice that fell from the maze were excluded from the statistical analyses.

#### Pavlovian conditioned fear

Mice were initially placed into the test chamber and undisturbed for 5 min. Then, an 80 dB (2.8 kHz) white noise was presented for 30 s as the conditioned stimulus (CS) followed by a mild (2 s, 0.75 mA) foot-shock, which served as the unconditioned stimulus (US). After 2 min, another CS-US pair was presented. The mouse was removed after 30 s and returned to its home cage. Responses such as running, jumping, and vocalizing in response to the shock were recorded. 24 h later, the mice were returned to the test chamber and freezing behavior was recorded for 5 min (context test). One hour later, the mouse was placed in the same chamber but in which environmental and contextual cues were changed for this auditory CS test: A black Plexiglas triangular insert was placed in the chamber to alter its shape and spatial cues, red house lights replaced the white house lights, the wire grid floor was covered with white Plexiglas, and 5% acetic acid was placed in the chamber to alter the smell. There were two phases during the auditory CS test; in the first phase (pre-CS), freezing was recorded for 3 min before presenting the CS. In the second phase, the auditory CS was turned on for 30 s and freezing was recorded for another 3 min. The number of freezing episodes was converted to a percentage freezing value.

#### Direct interaction test

The test animals were single-housed undisturbed in their home cage with bedding, food, and water for 48 h before the testing. The tails were label with a marker. For each test animal, a non-familiar, non-littermate wild-type C57BL/6 intruder was selected. The intruder was of similar age but always lighter than the test animal. The day of the experiment, the animals were taken to the room and left undisturbed for 30 min. The lid of the cage was removed, and the animals left in their home cage alone and videotaped for 10 min for the undisturbed period. Next an object (Eppendorf 1.5-ml tube) was put in the middle of the cage (same position for all the test mice), and the animals filmed for another 10 min. Then, the object was removed, and an intruder was added into the chamber and videotaped for another 10 min. In the first 10 min, the amount of time and frequency that the test animal spent self-grooming, digging, showing ambulatory movement, rearing, or jumping was measured. In the next 10 min, the interaction with the object was also measured (biting, pushing, sniffing, etc.). In the last 10 min, the time and frequency of tail sniffing/or following, face sniffing, mounting, wrestling, huddling, and allogrooming was also measured.

### X-gal staining and immunofluorescence

Mice were perfused with PBS, and 4% paraformaldehyde and brains were immediately dissected and fixed for 2–3 h at 4°C with 4% PFA, 2 mM MgCl2. Coronal sections (40 μm) were cut with a cryostat (Leica). Brain sections were kept in PBS with 2 mM MgCl2 at 4°C before staining. Free floating coronal sections were incubated overnight in X-gal staining solution (0.02% NP40, 0.01% sodium deoxycholate, 2 mM Mg Cl2, 5 mM K3Fe(CN)6, 5 mM K4Fe(CN)6, 0.5 mg/ml X-gal in PBS). Sections were then washed with PBS twice and then incubated with blocking buffer (2% goat serum, 0.03% Triton-X-100) and primary antibodies overnight (Rabbit anti-GFAP 1:500, Abcam Ab7260; mouse anti-NeuN 1:500, Chemicon MAB377). After 3 washes with PBS, secondary antibodies were added (1:1000 Goat anti-mouse Alexa Fluor568 and Goat anti-rabbit Alexa Fluor488, Invitrogen) and incubated for 1 h at 4°. Section were washed 3× in PBS and stained with DAPI. All the sections were mounted with Dako fluorescence mounting medium (Dako). Confocal images were acquired using a LSM710 Zeiss confocal microscope.

### Bone and cartilage staining

Four-month-old mice were dissected to remove all the skin, viscera, and adipose tissue. Bone and cartilage was fixed with 95% ethanol for 4 days, then placed in acetone to remove the fat for 1 day, following by 7 days staining at 37°C with 10 ml of staining solution (0.5 ml of 0.3% alcian blue 8GX (Alfa Aesar) in 70% ethanol, 0.5 ml alizarin red S (Alfa Aesar) in 95% ethanol, 0.5 ml glacial acetic acid, and 8.5 ml of 70% ethanol). After staining, the specimens were washed with abundant tap water for 5 min, cleared with 1% KOH for 3 days, and placed in 20% glycerin with 1% KOH for 5 days. The specimens were passed to 80% glycerin for several days and then stored in 100% glycerin.

### Mouse cortical cultures

E16.5 C57BL/6 mouse embryo cortices were dissected in 4.5 ml of Ca- and Mg-free Hank's balanced salt solution buffered with 10 mM HEPES pH 7.3 (CMF-HBSS) (Hyclone) and dissociated by adding 0.5 ml of 2.5% trypsin (Gibco-Life technology), and incubated for 15 min at 37°C. Trypsin treatment was terminated with three 5-min washes in CMF-HBSS. Cells were dissociated with a flame-narrowed Pasteur pipette. Neurons were seeded at a density of 7.5 × 10^5^ per well on 6-well plates. The plates were pre-coated overnight with poly-L-lysine (Sigma) in water and washed three times with water before use. Neurons were plated in MEM supplemented with 0.4% glucose and 10% fetal bovine serum. 24 hours later, the medium was changed to Neurobasal Medium containing B27 supplement (2%; Invitrogen), penicillin–streptomycin (50 μg/ml penicillin, 50 U/ml streptomycin, Sigma), and glutamine (1 mM, Sigma) (NBM). Half of the media was changed every 7 days.

### Time-lapse imaging of neurite outgrowth

Neurons were plated, and cellular morphology was recorded and quantified every 3 h for 48 h using an IncuCyte live-cell imaging system (Essen Instruments, Ann Arbor, MI) located within the incubator. Cells were imaged under phase. Analysis was performed using Incucyte's NeuroTrack software. The growth rate of neurites in each well was obtained by measuring the length covered by neurites and expressed as mm/mm^2^. The length and branch points of the neurites were normalized to the cell bodies.

### Cell culture and luciferase reporter gene assay

Neuro-2a (N2A, ATCC# CCL-131), HEK-293 (ATCC# CRL-1573), and NIH-3T3 cells were maintained in Dulbecco's modified Eagle's medium (Cellgro, Mediatech, Inc) and supplemented with 10% fetal bovine serum (Gibco, Life Technologies), penicillin (100 U/ml), and streptomycin (100 μg/ml) (Gibco, Life Technologies) at 37°C with 5% CO_2_ until 95% confluence was attained. GAL4-BD fusions with different versions of mouse MBD5 were co-transfected with the luciferase reporter plasmid pFR-Luc (Agilent Technologies). The vector pSV-β-Galactosidase (Promega Corporation) was also co-transfected for normalization purposes. After 48 h post-transfection, the cells were lysed and the luciferase activity was measured with Luciferase Assay kit (Agilent Technologies) according to manufacturer's instructions. Each experiment was repeated independently at least three times, and luciferase activity (Turner BioSystems 20/20n, Promega Corporation) and β-Galactosidase activity (microassay protocol, Agilent Technologies) were measured in duplicates.

### Statistical analysis

All the experimental data were analyzed using the independent samples *t*-test except for the viability that was analyzed utilizing the *chi-*square statistic test and the direct interaction test which was analyzed by Mann–Whitney *U*-test. Quantitative data are presented as mean ± SEM.

**The paper explained**

**Problem**

2q23.1 microdeletion syndrome is a rare genetic disease that causes intellectual disabilities, motor delay, autistic-like behaviors, and a distinctive craniofacial phenotype. Descriptive studies in humans suggest that haploinsufficiency of a single gene, methyl-CpG-binding domain protein 5 *(MBD5)*, causes the disease. An animal model is needed to confirm this hypothesis and unravel the mechanism underlying the phenotype of this disease.

**Results**

We generated and characterized a 2q23.1 microdeletion syndrome mouse model by disrupting the *Mbd5* gene. These mice have striking similarities in the phenotype compared to human carriers of the disease including abnormal social behavior, cognitive impairment, and motor and craniofacial abnormalities. In addition, cortical neuronal cultures allowed the detection of a deficiency in neurite outgrowth implicating Mbd5 in normal neuronal function.

**Impact**

The generation of this animal model confirms the causal role of MBD5 in the 2q23.1 microdeletion syndrome and indicates that neuronal dysfunction is responsible for the observed phenotype. A complete elucidation of the mechanism causing the phenotype observed in this syndrome not only will be important for the treatment of this rare disease but will provide light into common disorders that have similar phenotypes like autism and intellectual disabilities.

## Supporting Information

for this article is available online: http://embomolmed.embopress.org
