# Supplementary material for: Disruption of Mbd5 in mice causes neuronal functional deficits and neurobehavioral abnormalities consistent with 2q23.1 microdeletion syndrome
Source: EMBO Mol Med. 2014 Jul 7;6(8):1003–15. doi: 10.15252/emmm.201404044 (PMC4154129; doi:10.15252/emmm.201404044)
Supplement: Supplementary file 1 [file emmm0006-1003-sd1.pdf]

Supplemental figures for "Disruption of Mbd5 in mice causes neuronal functional deficits and neurobehavioral abnormalities consistent with 2q23.1 Microdeletion Syndrome."

Content:

Figure S1. The β geo gene-trap cassette from pU-21B vector was inserted into the second intron of *Mbd5*

Figure S2. Pattern of expression of mouse Mbd5

Figure S3. Reduced abdominal fat and nasal bone deformation in *Mbd5*$^{+/GT}$ mice

Figure S4. *Mbd5*$^{+/GT}$ mice have normal locomotor activity and anxiety-like behavior

Figure S5. Increased frequency of self-grooming and normal olfaction in *Mbd5*$^{+/GT}$ mice

Figure S6. Localization of transfected Mbd5 in mouse NIH-3T3 cells

Supplemental Table 1. Sequences of the primers used in the manuscript

Supplemental figure legends

Fig S1

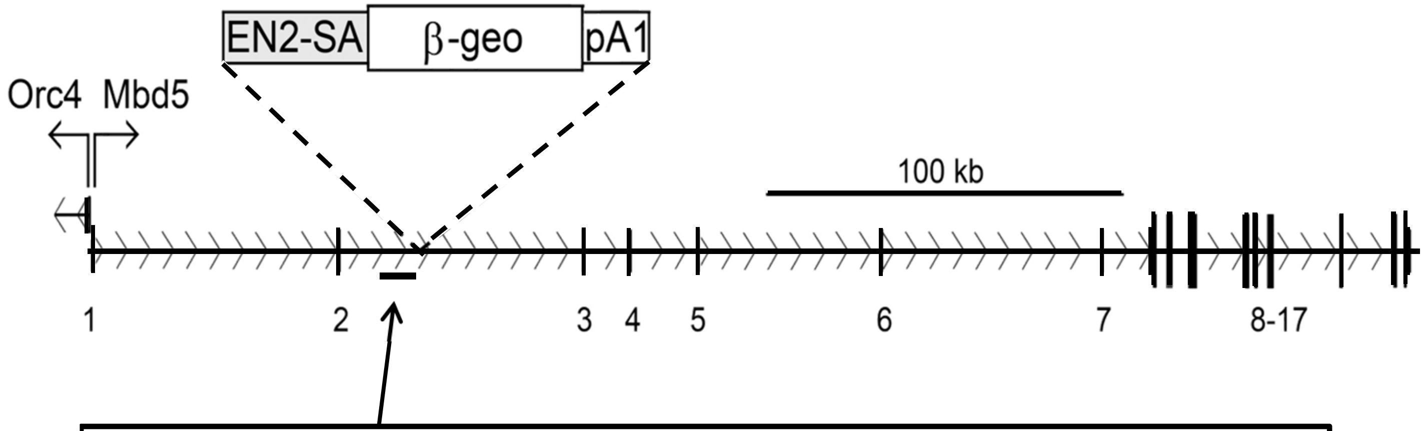

5'Race product from Gene trap allele: Mbd5$^{Gt(Ayu21\text{-}B205)Imeg}$

Trap vector: pU-21B Size 9399 bp
Vector integration sites were determined by performing BLAST analysis of the 5'RACE using the UCSC database: (chr2:49,081,787).
Result of the 5'RACE sequence: underlined align with the *Mbd5* gene, italic sequence align with the trap vector.

cactattattgtgtgagttgtagtgtgttctttattagagtttccttaatgaatgtgggatgcccttgcatttga
agcacagatattcacaattgagagttcatcttggaagattttctttagtgagtatgaagtgcacctccttgt
ctttttgataactttgggttggaagttgattttattttatattagaatggctactccagcttgtttcttcagagtg
tttgcttggaaaattgttttccagccttttcctctgaggtagggtatgtctttgtccctgaggtgggtttcctgt
atgtaggaaaatgttcagtcctgtttatgtagccaggctgttagtctaagtctttttattggggaattgagtc
cattgatattaagagatactaaggaaaagtgagtgttgcttcctatttttgttgttagag*tcttgtcctagaa*
gcctcactggccaggtgtaagccaggtcgtgggtgccgagccctgctccctcatcctcagcatggat
gtgaagaggactgtatggcgtgcgggtgtgtgtgaccgtgggtacacttaaaacaccgggtttggatc
tgcat

Fig S2

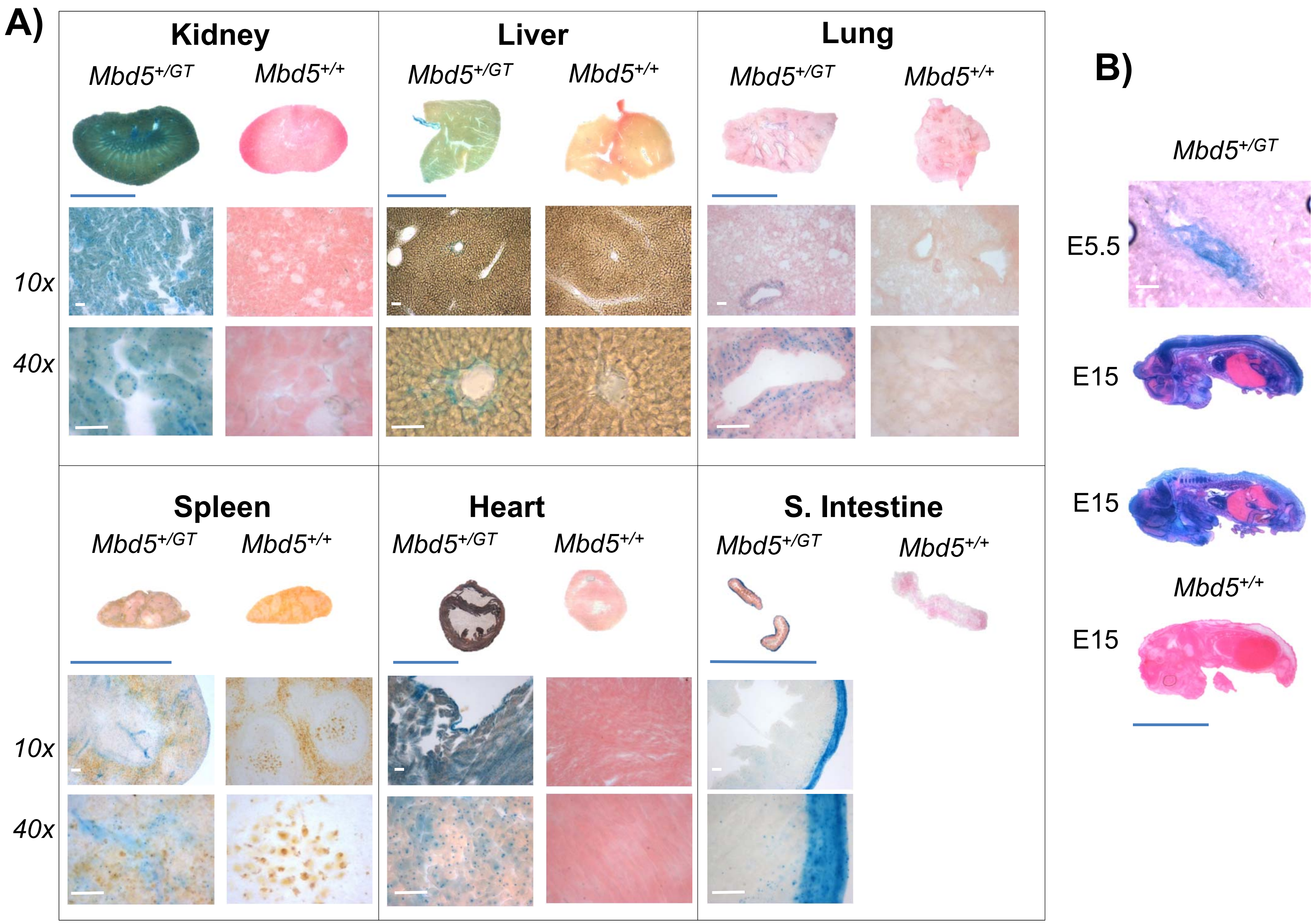
A)
Kidney
Liver
Lung
Mbd5+/GT
Mbd5+/+
10x
40x
Spleen
Heart
S. Intestine
B)
Mbd5+/GT
E5.5
E15
E15
Mbd5+/+
E15

Fig S3

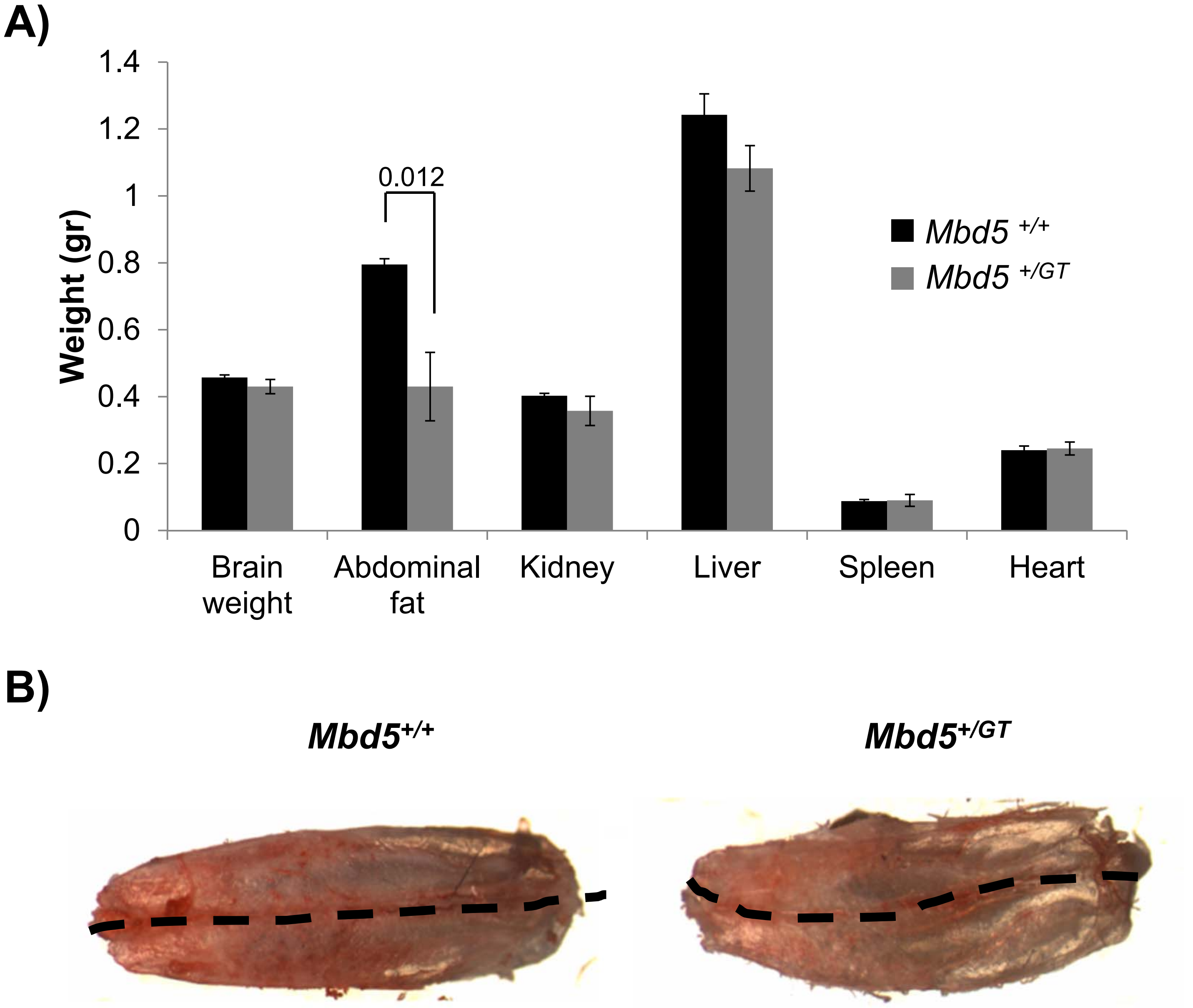

A)
1.4
1.2
1
0.8
0.6
0.4
0.2
0
Weight (gr)
0.012
Mbd5 +/+
Mbd5 +/GT
Brain weight
Abdominal fat
Kidney
Liver
Spleen
Heart
B)
Mbd5+/+
Mbd5+/GT

Fig S4

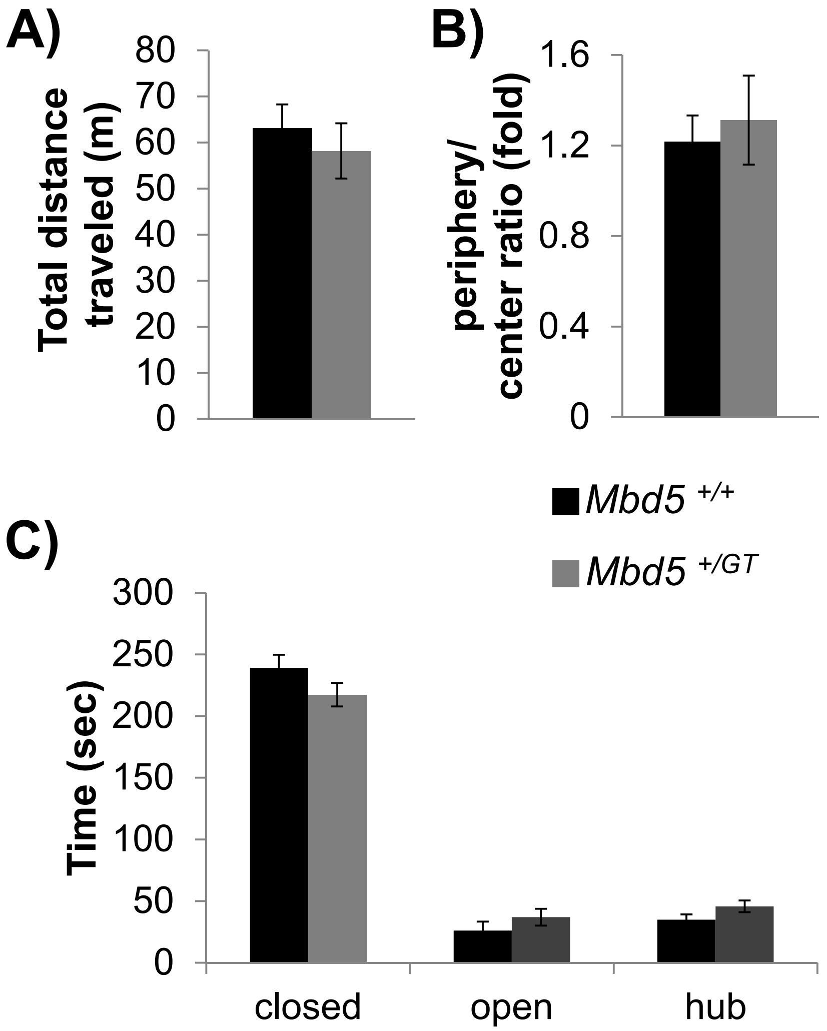
A)
Total distance traveled (m)
0
10
20
30
40
50
60
70
80
B)
periphery/ center ratio (fold)
0
0.4
0.8
1.2
1.6
Mbd5 +/+
Mbd5 +/GT
C)
Time (sec)
0
50
100
150
200
250
300
closed
open
hub

Fig S5

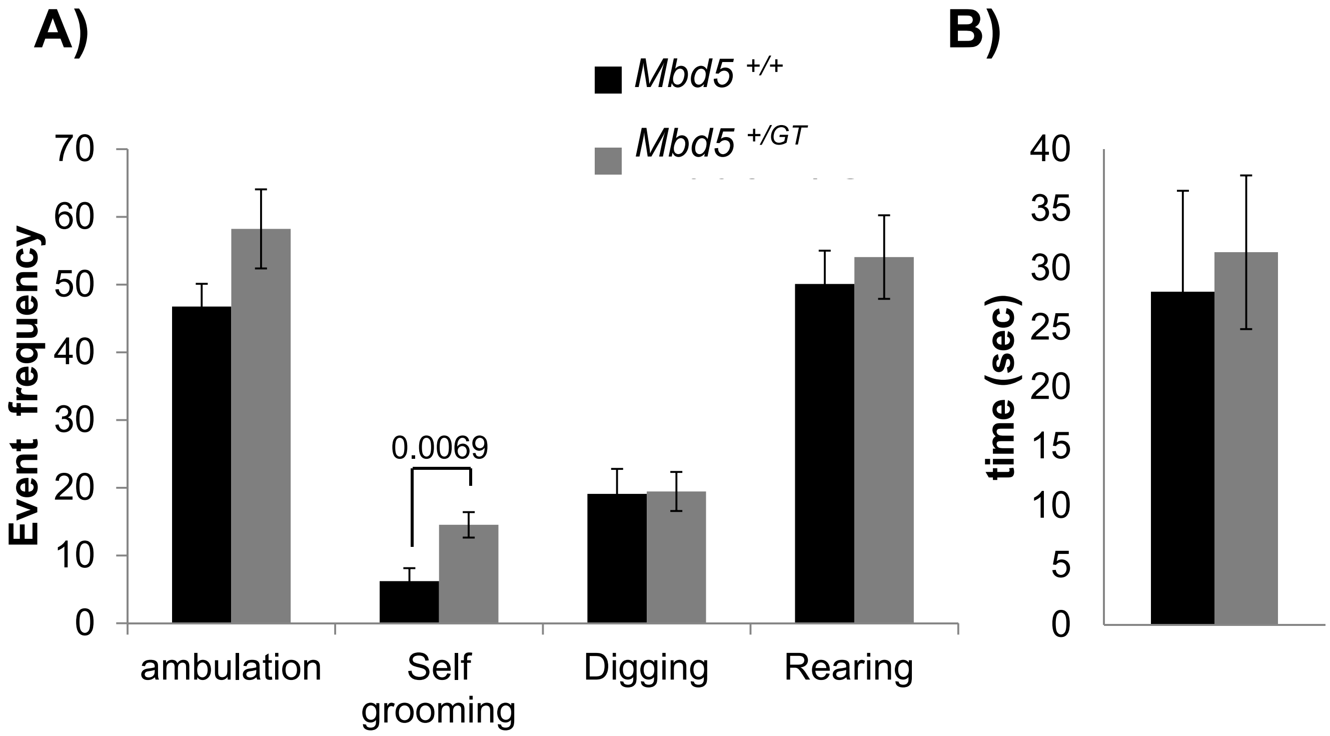
A)
B)
Mbd5 +/+
Mbd5 +/GT
Event frequency
0
10
20
30
40
50
60
70
0.0069
ambulation
Self grooming
Digging
Rearing
time (sec)
0
5
10
15
20
25
30
35
40

Fig S6

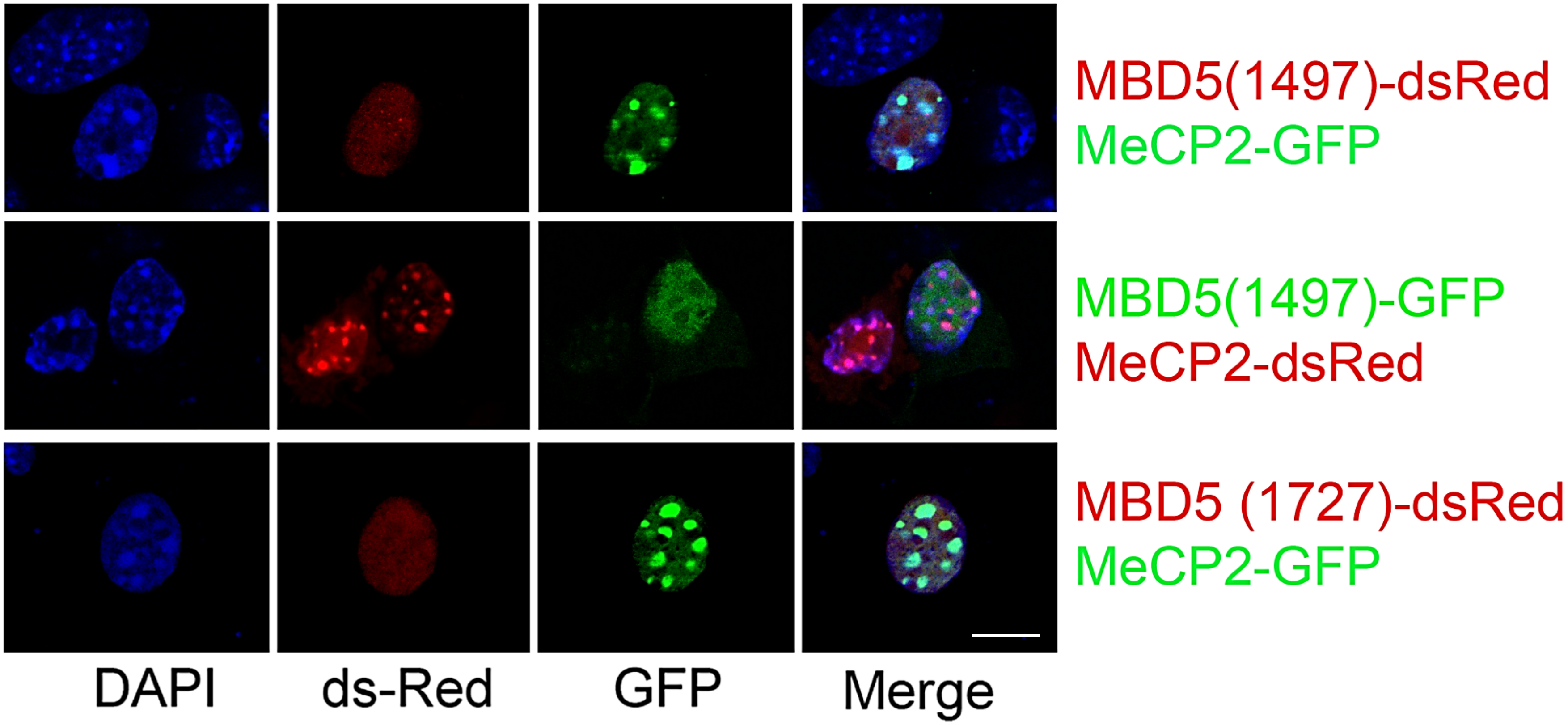
MBD5(1497)-dsRed
MeCP2-GFP
MBD5(1497)-GFP
MeCP2-dsRed
MBD5 (1727)-dsRed
MeCP2-GFP
DAPI
ds-Red
GFP
Merge

Supplemental Table 1

Primer name	Primer sequence
GapdhF	ACCCAGAAGACTGTGGATGG
GapdhR	CACATTGGGGGTAGGAACAC
Gsk3bF	TGGCAGCAAGGTAACCACAG
Gsk3bR	CGGTTCTTAAATCGCTTGTCCTG
Sgk1F	CGCCAAGTCCCTCTCAACAA
Sgk1R	TGCCCTTTCCGATCACTTTC
c-fosF	ACCATGATGTTCTCGGGTTTCAA
c-fosR	GCTGGTGGAGATGGCTGTCAC
Tet2F	GCTGAGCCAAAAGAGGAAGAG
Tet2R	TGAGGGTGACCACCACTGTA
CrebF	TCAGCCGGGTACTACCATTC
CrebR	TTCAGCAGGCTGTGTAGGAA
BdnfF	CATATCGGCCACCAAAGACT
BdnfR	GTCCGCACACCTGGGTAG
ArcF	AGCAGCAGACCTGACATCCT
ArcR	GGTGTCATTCTCCTGGCTCT
Mbd5 p2	TCGGATCCTAACTAAATCAAAATG
Mbd5 pT	GTAATGGGATAGGTTACGTTGGTGTAG
Mbd5 p4	CACCACACAGCCTGCAATAG
Mbd5 p13F	TCTCCAGGGACACCAGAATC
Mbd5 p15R	GCGGCTGTAGGCTTCTAAAC
Orc4F	GCACATGCAGAGTGTCTTTCA
Orc4R	TTTTCACTCACTTCTATTTCCATGA
Mbd5 p10F	CAGTCCTGGAGGAGGAACAA
Mbd5 p11R	TTCACAATGGGGAAAGGAAC
Mbd5 p12F*	GGCAGCACTGACTGTCTCAA
Mbd5 p12F	TCAACAGCATCTCCTAAACCAG
Mbd5 p13R	TGCTGATTTGGAGGAAACATC

Supplemental figure legends

Figure S1. The β geo gene-trap cassette from pU-21B vector was inserted into the second intron of *Mbd5*: Schematic of the *Mbd5* gene with the insertion of the β geo gene-trap cassette. Numbers correspond to the exons of Mbd5. Sequence from the 5'-inverse PCR product from the gene trap allele is in the box. Vector integration site was determined by BLAT search of the 5'-inverse PCR sequence using the UCSC genome browser.

Figure S2. Pattern of expression of mouse Mbd5. 30 μm cryostat sections from (a) 1 month old mice kidney, liver, lung, spleen, heart and small intestine and (b) from whole mount mouse embryos at E5.5 and E15 from $Mbd5^{+/+}$ and $Mbd5^{+/GT}$ male mice were processed for X-gal staining. Transmittance images of magnified regions are shown. (n=2 $Mbd5^{+/+}$, n=4 $Mbd5^{+/GT}$). White scale bar: 40μm; Blue scale bar: 5mm.

Figure S3. Reduced abdominal fat and nasal bone deformation in $Mbd5^{+/GT}$ mice. (a) Average weight of the brain, abdominal fat, kidney, liver, spleen and heart of male $Mbd5^{+/+}$ and $Mbd5^{+/GT}$ mice measured at 4 months of age (4 $Mbd5^{+/+}$ and 4 $Mbd5^{+/GT}$; Student's T test; unpaired, 2 tailed distribution, p values are displayed above the bars in the figure). (b) Top view of the dissected nasal bone of male $Mbd5^{+/+}$ and $Mbd5^{+/GT}$ mice at 1 month of age. The intermittent line highlights the curvature of the midline and the lack of symmetry in nasal bones of $Mbd5^{+/GT}$ mice.

Figure S4. ***Mbd5***$^{+/GT}$ **mice have normal locomotor activity and anxiety-like behavior.** (a) Locomotor activity measured in the open field do not show significant difference between Mbd5$^{+/+}$ and *Mbd5*$^{+/GT}$ male mice, (b) the periphery/center distance ratio in the open field and (c) the time spent in open arm of the elevated plus maze are indicators of anxiety-like behavior and do not show any significant difference between *Mbd5*$^{+/+}$ and *Mbd5*$^{+/GT}$ male mice (Student's T test, unpaired, 2 tailed distribution, p values are displayed above the bars in the figure, n= 9 *Mbd5*$^{+/+}$ and 16 *Mbd5*$^{+/GT}$, 9 weeks old mice).

Figure S5. Increased frequency of self-grooming and normal olfaction in ***Mbd5***$^{+/GT}$ **mice.** (*a*) Evaluation of frequency of activities of unperturbed male mice observed over 10 min in their own cage. Bars represent frequency of the events of the indicated activity. Age of mice was 3 months. (n=9 *Mbd5*$^{+/+}$ and 14 *Mbd5*$^{+/GT}$, Mann-Whitney U test, p values are displayed above the bars in the figure) (*b)* the time required to find a hidden food pellet in *Mbd5*$^{+/+}$ and *Mbd5*$^{+/GT}$ was the same in the odor test.

Figure S6. Localization of transfected Mbd5 in mouse NIH-3T3 cells. Mbd5 and the heterochromatic marker MeCP2 fused to GFP or dsRed at the N-terminus were transfected into NIH-3T3 cells. DAPI was used for nuclei acid staining (blue). The images were taken with a confocal LSM 710 microscope from Zeiss. Scale Bar = 10µm.

Supplementary Table 1. Sequences of the primers used in the manuscript.
