# Supplementary material for: Disruption of Mbd5 in mice causes neuronal functional deficits and neurobehavioral abnormalities consistent with 2q23.1 microdeletion syndrome
Source: EMBO Mol Med. 2014 Jul 7;6(8):1003–15. doi: 10.15252/emmm.201404044 (PMC4154129; doi:10.15252/emmm.201404044)
Supplement: Supplementary file 2 [file emmm0006-1003-sd2.pdf]

EMBO Molecular Medicine

Disruption of Mbd5 in mice causes neuronal functional deficits and neurobehavioral abnormalities consistent with 2q23.1 Microdeletion Syndrome.

Vladimir Camarena, Lei Cao, Clemer Abad, Abrams Alexander, Yaima Toledo, Kimi Araki, Masatake Araki, Katherina Walz and Juan I. Young

Corresponding author: Juan Young, University of Miami

Review timeline:

Submission date:	11 March 2014
Editorial Decision:	10 April 2014
Revision received:	14 May 2014
Editorial Decision:	21 May 2014
Revision received:	28 May 2014
Accepted:	02 June 2014

Transaction Report:

(Note: With the exception of the correction of typographical or spelling errors that could be a source of ambiguity, letters and reports are not edited. The original formatting of letters and referee reports may not be reflected in this compilation.)

Editor: Céline Carret

1st Editorial Decision 10 April 2014

Thank you for the submission of your manuscript to EMBO Molecular Medicine. We have now heard back from the three referees whom we asked to evaluate your manuscript.

You will see that they all find the topic of your manuscript interesting but they feel that the data need to be strengthened by better images, better explanations, and better justified statistics and they make constructive suggestions for improvement. Referees 1 and 2 are concerned about the KCl depolarisation experiment, and this should be addressed. They all also suggest a thorough proof-reading of the manuscript prior to resubmission. Overall, should you be able to address these criticisms in full, we would be happy to consider a revised manuscript.

Please note that it is EMBO Molecular Medicine policy to allow a single round of revision in order to avoid the delayed publication of research findings. Consequently, acceptance or rejection of the manuscript will depend on the completeness of your responses included in the next version of the manuscript.

EMBO Molecular Medicine has a "scooping protection" policy, whereby similar findings that are published by others during review or revision are not a criterion for rejection. Should you decide to submit a revised version, I do ask that you get in touch after three months if you have not completed it, to update us on the status.

Please also contact us as soon as possible if similar work is published elsewhere. If other work is published we may not be able to extend the revision period beyond three months.

I look forward to seeing a revised form of your manuscript as soon as possible.

***** Reviewer's comments *****

Referee #1 (Comments on Novelty/Model System):

Haploinsufficiency of MBD5 is thought to be causative for the neurological deficits of 2q23.1 microdeletion syndrome, but no mouse model of this deletion has been made previously and nearly nothing is known about the cellular functions of MBD5. This manuscript develops and provides characterization of a mouse model for partial loss of MBD5 function that will be very useful for future investigation of this disorder. This manuscript also provides key evidence of MBD5 expression and function that will advance cellular understanding of its functions.

Referee #1 (Remarks):

This is a well-written manuscript reporting the development and behavioral characterization of a gene trap mouse model for the role of the methyl-DNA binding protein MBD5 in 2q23.1 microdeletion syndrome. In addition to demonstrating that the mouse model recapitulates the behavioral features of the syndrome, suggesting it is useful for studying the pathophysiology of this disease, the manuscript provides key evidence of MBD5 expression and function that advance fundamental understanding of this poorly understood methyl-DNA binding protein. The data are logically presented and the story is very novel.

I have only one significant concern, which is about Figure 7 - the data on expression of Gsk3b and Tet2 mRNA after KCl depolarization of +/+ and +/GT neurons. No reasonable explanation is given why these genes were assayed. These are not activity-regulated genes and a questionable difference is seen at only one time point. Overall there is no difference if all time points are considered (and ANOVA, not t test, would be required to determine that there is an effect of treatment over time on either genotype followed by a need to see and interaction between treatment and genotype, which I suspect the authors would not see). The much stronger data to suggest MBD5 regulates transcription are the Gal4 data in Figure 9. I suggest that the authors should remove Figure 7 from the manuscript, as it only detracts.

I have only one minor concern, which is the misspelling of MeCP2 ("MePC2") on pg. 4.

Referee #2 (Remarks):

Given the genetic evidence of a partial or full deletion of the methyl-CpG-binding domain protein 5, MBD5, in 2q23.1 microdeletion syndrome, Camarena et al set out to test the causal role of Mbd5 in the syndrome. They generated and characterized an Mbd5 gene trap mouse model in which the expression of Mbd5 is disrupted. They found that most of Mbd5 mRNAs are ablated by the gene trap and homozygous Mbd5 GT/GT mice are perinatal lethal. Using a series of behavioral tests, they demonstrate that Mbd5+/GT heterozygotes recapitulate many of the hallmark phenotypes observed in 2q23.1 deletion carriers, including abnormal social behavior, cognitive impairment, motor dysfunction and craniofacial abnormalities. They also show that cultured Mbd5+/GT cortical neurons have reduced neurite outgrowth and branching compared to controls. Furthermore, they observed that Mbd5 is localized in the nucleus and increases luciferase activity in a reporter assay. Together this study provides strong evidence supporting the causal role of MBD5 in the 2q23.1 microdeletion syndrome. The proposed molecular function or mechanism for Mbd5, however, is not convincing. But my enthusiasm is not dampened to support the publication of this manuscript in EMM, primarily because of the biological significance and clinical relevance of this study. Additional comments are listed as following:

1) As the authors cited, an independent Mbd5-null mouse model was developed and published (Du et al, 2012). The published study, however, did not report any behavioral phenotypes, except survival curve. The authors should compare their findings with previously reported ones and discuss

any similarities and discrepancies in their manuscript.

2) The nuclear localization of the two MBD5 isoforms observed in this study is different from a previously published report as well (Laget et al, 2010). In that study, the authors reported MBD5 co-localizes with heterochromatin, in contrast to the localization pattern provided by this study. Again, discussion is needed in the manuscript to help readers understand the discrepancies.

3) The authors attempted to identify gene expression changes and cellular morphology changes using cultured cortical neurons in vitro and depolarizing them with KCl. I don't think this experiment is well justified. Would in vitro culture and KCl depolarization produce more variables? In addition, characterizing the molecular function of MBD5 or identifying a mechanism by which loss-of-function of Mbd5 leads to the behavioral phenotypes is a tall order to achieve in one study. In my opinion, including a poorly characterized mechanism weakens the study at the current form.

4) The authors concluded that all 5 mRNA isoforms of Mbd5 were detected by RT-PCR. Have they sequenced these PCR products to confirm the identity?

5) X-gal staining images presented in Fig. 3A and IF images in 3B are either in poor quality due to technical reasons or "real"? I recommend the authors solve their technical issues if that's the case.

6) The GFP images in Fig. 9 are poor as well likely due to technical reasons. These need to be improved.

7) Have the authors measured the nucleus or cell body size in Mbd5 GT/+ neurons?

8) Proof read of the manuscript is highly recommended.

Referee #3 (Remarks):

Camerena et al.'s paper "Disruption of Mbd5 in mice causes neuronal functional deficits and neurobehavioral abnormalities consistent with 2q23.1 Microdeletion Syndrome" details the group's work generating a murine model for 2q23.1 microdeletion syndrome. The work indicates that disruption of the Mbd5 gene in mice decreases the amount of functional mRNA and protein and closely recapitulates the human disorder both physically and behaviorally. The paper is extremely well-written, concise and to the point.

My critiques are few.

Stylistically there could be some changes to be made:

1. More effort should be made in the introduction to discuss the genes involved with deletion locus found the 2q23.1 Microdeletion Syndrome and the background on why these genes are thought to not be playing a role in the phenotype compared to Mbd5.

2. More effort should be made in the Discussion section to compare the paper by Du et al. with this paper. Why did there heterozygotes not have the same phenotype etc.

3. Fig 2B the x-axis needs to have a better label such as "primer pairs"

4. "perinataly" is misspelled and perhaps is not an actual word. MECP2 is misspelled on several occasions and the "GT " of gene trap is often misspelled as "TG".

5. In the B-gal analysis of the heterozygote brain expression I do not believe there is any comment about the anatomy of the brain. I believe it is normal, but in a mouse that is an affected heterozygote in many ways it is important to include a paragraph discussing macro- and micro-anatomy of the brain and that the Mbd5 dosage effect does not extend to the anatomy.

6. Was any effort put into looking at B-geo expression in adipose? This would be interesting to determining whether this is an issue of local adipose tissue dysfunction or general decreased ability to store energy (in the form of fat).

7. It might be worthwhile to state in the discussion that the in vitro data on transcriptional activity is done indirectly via the GAL4 DNA binding domain fusion protein and are not direct evaluation of activity from Mbd5 interacting with a known "Mbd5 promoter". In the context of Gal4 hybrids this transactivation could be real or just an artifactual effect while in the proximity of the DNA promoter... and not it's natural function.

8. In the discussion section, within a sentence of each other there is contradictory statements regarding the mice and autistic spectrum. On one hand the repetitive grooming is said to be autistic behavior, but two sentences later the animals increased social behavior is decidedly NON-autistic... some better wording with regards to the behavioral phenotypes may be appropriate. The mice cannot be both autistic and non-autistic at the same time, perhaps better to include a sentence about this in the discussion.

1st Revision - authors' response 14 May 2014

We appreciate the thoughtful comments and suggestion of the reviewers. In particular, we are pleased that they all found our manuscript "extremely well-written, concise and to the point", "very novel", and of "biological significance and clinical relevance". We have made every effort to address all reviewers' comments, as you could see in our detailed rebuttal.

Point by point rebuttal:

Reviewer #1:

*1- **Reviewer comment**: "I have only one significant concern, which is about Figure 7……… I suggest that the authors should remove Figure 7 from the manuscript, as it only detracts."*

Authors' answer: After careful consideration, we concur with the reviewer that Figure 7 and its accompanying data are not very informative and have therefore removed them, as suggested by the reviewer.

*2- **Reviewer comment:** "I have only one minor concern, which is the misspelling of MeCP2 ("MePC2") on pg. 4."*

Authors' answer: We thank the reviewer for catching the typo. The misspelling has been fixed.

Reviewer #2:

***Reviewer general comment:** "The proposed molecular function or mechanism for Mbd5, however, is not convincing".*

Authors' answer: Please see response to Reviewer #1 comment 1. We have removed figure 7 and the corresponding non convincing data.

*1- **Reviewer comment**: "The authors should compare their findings with previously reported ones and discuss any similarities and discrepancies in their manuscript."*

Authors' answer: We have added the following phrase in the discussion section: "The heterozygous MBD5-null animals were described as grossly normal, however they do not report their neurobehavioral phenotype (Du et al. 2012).". Page 14.

*2- **Reviewer comment**: "The nuclear localization of the two MBD5 isoforms observed in this study is different from a previously published report as well (Laget et al, 2010)……Again, discussion is needed in the manuscript to help readers understand the discrepancies.*

Authors' answer: We modified the text as follows: "Laget et al reported that human MBD5 formed nuclear puncta when transfected into mouse cells that coincided with mouse heterochromatin foci (Laget et al. 2010). However, we observed MBD5 exclusion from heterochromatic foci (Figure 8C). To further test if MBD5 localizes to heterochromatin, we co-transfected N2A and NIH-3T3 cells with MBD5 along with MeCP2, which has a bona fide heterochromatic localization. We observed mostly non overlapping localization of these two proteins in both cells types, suggesting that mouse MBD5 does not bind heterochromatin in cultured cells (Figure 8C and Supplemental figure S6)." Page 13, Results section.

Upon the suggestion of the reviewer, we transfected our fully sequenced mouse MBD5 constructs into the same mouse NIH-3T3 cells in which Laget et al observed heterochromatic localization of **human** MBD5, and again saw no colocalization of MBD5 (neither the mouse isoform equivalent to the human MBD5 they used, nor an alternative larger mouse isoform) with heterochromatin or MeCP2 (these data are added as a new supplemental figure S6). The different results might be explained by the different origins of the MBD5 cDNAs: human in the case of Laget and mouse in the case of this manuscript, or to the localization of the fused fluorescent protein (N-terminus in their case, while in ours both the ds-Red and GFP are fused at the C-terminus).

*3- **Reviewer comment**: "… characterizing the molecular function of MBD5 or identifying a mechanism by which loss-of-function of Mbd5 leads to the behavioral phenotypes is a tall order to achieve in one study. In my opinion, including a poorly characterized mechanism weakens the study at the current form."*

Authors' answer: Please see response to Reviewer #1 comment 1. We have removed figure 7 and the corresponding poorly characterized mechanistic data.

*4- **Reviewer comment: "**The authors concluded that all 5 mRNA isoforms of Mbd5 were detected by RT-PCR. Have they sequenced these PCR products to confirm the identity?"*

Authors' answer: Yes, the RT-PCR products were sequenced. We have added "followed by confirmatory sequencing" to the original phrase: "We analyzed the expression of Mbd5 isoforms by RT-PCR in mouse tissues at different developmental time points". The final phrase reads: "We analyzed the expression of Mbd5 isoforms by RT-PCR, followed by confirmatory sequencing, in mouse tissues at different developmental time points". Page 5.

*5- **Reviewer comment: "**X-gal staining images presented in Fig. 3A and IF images in 3B are either in poor quality due to technical reasons or "real"? I recommend the authors solve their technical issues if that's the case."*

Authors' answer: When converted to pdf, the images significantly lost quality. However, we did not realize this fact until we saw the reviewer's comments. We apologize for the poor quality. This resubmission is accompanied by high quality images.

*6- **Reviewer comment: "**The GFP images in Fig. 9 are poor as well likely due to technical reasons. These need to be improved."*

Authors' answer: See above response to Reviewer's comment 5.

*7- **Reviewer comment: "**Have the authors measured the nucleus or cell body size in Mbd5 GT/+ neurons?"*

Authors' answer: A preliminary comparison of the nucleus/body size in the hippocampous CA1 area in the in Mbd5 +/GT vs wild type did not detect any significant difference. However, at this point we can't rule out an effect in nuclear size at specific neurons due to decreased MBD5.

© EMBO

*8- **Reviewer comment:** Proof read of the manuscript is highly recommended.*

Authors' answer: We have asked a native English speaker to proofread the manuscript.

Reviewer #3:

*1- **Reviewer comment:** "... discuss the genes involved with deletion locus found the 2q23.1 Microdeletion Syndrome and the background on why these genes are thought to not be playing a role in the phenotype compared to Mbd5."*

Authors' answer: We have added the following text regarding other genes involved in the 2q23.1 locus: "Although the 2q23.1 locus contains several genes associated with genetic disorders such as ORC4, KIF5C, MMADHC, NEM2 and CACNB4, extensive analysis of the alignment of deleted regions in patients identified MBD5 as the single gene included in the smallest region of overlap, suggesting that genetic alterations of MBD5 cause features of 2q23.1 microdeletion syndrome." Page 14, Discussion section.

*2- **Reviewer comment:** ".... compare the paper by Du et al. with this paper. Why did there heterozygotes not have the same phenotype etc."*

Authors' answer: The paper from Du et al. did not perform a neurobehavioral characterization of the MBD5-null heterozygous. Please see response to Reviewer #2, comment 1.

*3- **Reviewer comment:** "Fig 2B the x-axis needs to have a better label such as "primer pairs""*

Authors' answer: We have incorporated this suggestion. X-axis of Fig 2B has been edited as suggested,

*4- **Reviewer comment:** ""perinataly" is misspelled and perhaps is not an actual word. MECP2 is misspelled on several occasions and the "GT " of gene trap is often misspelled as "TG"."*

Authors' answer: We thank the reviewer for catching the typos. All misspellings have been fixed.

*5- **Reviewer comment:** "In the B-gal analysis of the heterozygote brain expression I do not believe there is any comment about the anatomy of the brain. I believe it is normal, but in a mouse that is an affected heterozygote in many ways it is important to include a paragraph discussing macro- and micro-anatomy of the brain and that the Mbd5 dosage effect does not extend to the anatomy."*

Authors' answer: Overtly, the anatomy of the brain of MBD5+/GT mice looks normal. However, we don't feel comfortable affirming that macro- and micro-anatomy of the brain is normal without a systematic analysis and therefore choose not to discuss this issue.

*6- **Reviewer comment:** "Was any effort put into looking at B-geo expression in adipose? This would be interesting to determining whether this is an issue of local adipose tissue dysfunction or general decreased ability to store energy (in the form of fat).***"**

Authors' answer: we agree with the reviewer that this is an interesting point, however, we consider that it is out of the scope of this manuscript to decipher the origin of the decreased fat content.

*7- **Reviewer comment:** "It might be worthwhile to state in the discussion that the in vitro data on transcriptional activity is done indirectly via the GAL4 DNA binding domain fusion protein and are not direct evaluation of activity from Mbd5 interacting with a known "Mbd5 promoter". In the context of Gal4 hybrids this transactivation could be real or just an artifactual effect while in the proximity of the DNA promoter... and not it's natural function."*

Authors' answer: We have added the following text: "Supporting this suggestion, we observed that MBD5 has transcriptional activator functions in vitro when fused to a GAL4 DNA binding domain.

Although this activity was not directly evaluated with an endogenous Mbd5 promoter, our data is in agreement with a recent report (published while this manuscript was under revision) showing that MBD5 directly activates the transcription of the Fth1 promoter (Tao et al. 2014)." to the original phrase: "Supporting this suggestion, we observed that MBD5 has transcriptional activator functions in vitro."

8- Reviewer comment: *"In the discussion section, within a sentence of each other there is contradictory statements regarding the mice and autistic spectrum. On one hand the repetitive grooming is said to be autistic behaviour, but two sentences later the animals increased social behaviour is decidedly NON-autistic... some better wording with regards to the behavioural phenotypes may be appropriate. The mice cannot be both autistic and non-autistic at the same time, perhaps better to include a sentence about this in the discussion."*

Authors' answer: This reviewer raised an interesting point. Traditionally, autism was seen as the result of the co-occurrence of impairments in three behavioural domains, abnormal social interactions, communication deficits and repetitive behaviours. However, despite the general agreement about the diagnostic validity of the triad of impairments, the relative contribution of each of the major symptoms to the diagnosis of autism is currently a matter of debate. There are indications that the covariation between deficits in sociability, communication, and occurrence of repetitive behaviours remains modest, *suggesting a potential independence of these three domains* (F. Happe, A. Ronald, R. Plomin. Time to give up on a single explanation for autism. Nature Neuroscience, 9 (10) (2006), pp. 1218–1220.). In fact, a large number of mouse models of ASD exhibit a single or variable combinations of two of the endophenotypes of the triad (see examples in Silverman et al Nature Reviews Neuroscience 11, 490-502 (July 2010)). For example, oxytocin receptor knockout mice show robust decreases in reciprocal social interactions, and reduced levels of communication, but no changes in repetitive behaviours; whereas *Mecp2* mutant mice show enhancement of social behaviour and communication, and no changes in repetitive behaviours (Pobbe RL, Pearson BL, Blanchard DC, Blanchard RJ. Oxytocin receptor and Mecp2 308/Y knockout mice exhibit altered expression of autism-related social behaviours. Physiol Behav. 2012 Dec 5;107(5):641-8.). Thus, our observation of persistent interests in the MBD5+/Gt mice in the absence of diminished social interest is not surprising. Notably, specific deletion of MeCP2 in GABA-releasing neurons results in a phenotype similar to one observed for MDB5 mice: repetitive behaviours and enhancement of social interactions. Same results were obtained in mice with loss of MeCP2 in a subset of forebrain GABAergic neurons. These results, along with ours suggest that the increase in social interaction could also be a manifestation of repetitive behaviour. In summary, our interpretation of the data differs with the reviewer's view that "increased social behaviour is decidedly NON-autistic". The exaggerated social engagement of these mice – abnormal not only in its persistence but also in its characteristics (increased dominance behaviours) – is most probably another reflection of the insistence on sameness exhibited by these MBD5+/GT mice.

Per reviewer's suggestion, we modified the text by adding the phrase "The exaggerated social engagement of these mice – abnormal not only in its persistence but also in its characteristics (increased dominance behaviours) – is most probably another reflection of the insistence on sameness exhibited by the Mbd5+/GT mice." . Page 15, Discussion section.

Thank you very much again. We look forward to publication of our work in EMBO Molecular Medicine.

2nd Editorial Decision 21 May 2014

Thank you for the submission of your revised manuscript to EMBO Molecular Medicine. We have now received the enclosed reports from the referees that were asked to re-assess it. As you will see the reviewers are now globally supportive and I am pleased to inform you that we will be able to accept your manuscript pending editorial final amendments:

Please submit your revised manuscript within two weeks. I look forward to seeing a revised form of

your manuscript as soon as possible.

***** Reviewer's comments *****

Referee #1 (Comments on Novelty/Model System):

This model is both clinically relevant to 2q23.1 microdeletion syndrome and useful for understanding the cellular functions of MBD5.

Referee #1 (Remarks):

The authors have addressed all of my concerns. I think the novel findings in this manuscript will be of great interest to the readership of this journal.

Referee #2 (Remarks):

The revised manuscript has adequately addressed the questions I raised in the first round of review. It is acceptable in my opinion.

Referee #3 (Remarks):

I am happy with the way that the authors have addressed my concerns from the first draft.
